# Performance of deep learning algorithms to distinguish high-grade glioma from low-grade glioma: A systematic review and meta-analysis

**DOI:** 10.1016/j.isci.2023.106815

**Published:** 2023-05-05

**Authors:** Wanyi Sun, Cheng Song, Chao Tang, Chenghao Pan, Peng Xue, Jinhu Fan, Youlin Qiao

**Affiliations:** 1 Department of Cancer Epidemiology, National Cancer Center/National Clinical Research Center for Cancer/Cancer Hospital, Chinese Academy of Medical Sciences and Peking Union Medical College, Beijing, China; 2 School of Population Medicine and Public Health, Chinese Academy of Medical Sciences and Peking Union Medical College, Beijing, China; 3 Shenzhen Maternity & Child Healthcare Hospital, Shenzhen, China

**Keywords:** Oncology, Machine learning

## Abstract

This study aims to evaluate deep learning (DL) performance in differentiating low- and high-grade glioma. Search online database for studies continuously published from 1st January 2015 until 16th August 2022. The random-effects model was used for synthesis, based on pooled sensitivity (SE), specificity (SP), and area under the curve (AUC). Heterogeneity was estimated using the Higgins inconsistency index (I^2^). 33 were ultimately included in the meta-analysis. The overall pooled SE and SP were 94% and 93%, with an AUC of 0.98. There was great heterogeneity in this field. Our evidence-based study shows DL achieves high accuracy in glioma grading. Subgroup analysis reveals several limitations in this field: 1) Diagnostic trials require standard method for data merging for AI; 2) small sample size; 3) poor-quality image preprocessing; 4) not standard algorithm development; 5) not standard data report; 6) different definition of HGG and LGG; and 7) poor extrapolation.

## Highlights

• It is aimed to evaluate deep learning performance in glioma classification
• We found DL achieved high accuracy in glioma grading
• Heterogeneity was found in pooled analysis and still remained in subgroup analysis
• In different subgroups, the performance of DL suggested major limitations in this field

## Graphical abstract

**Figure:**
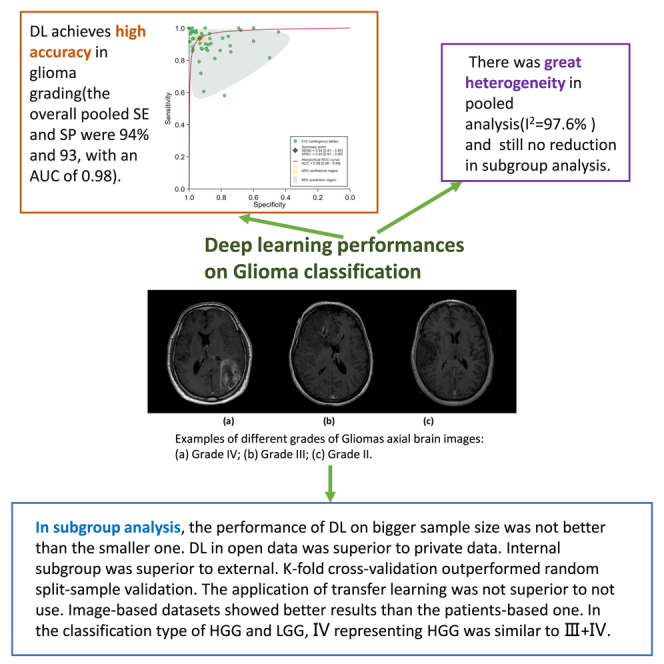

## Introduction

Glioma originates in the glial cells surrounding and supporting neurons in the brain and is the most common type of malignant brain tumor, representing approximately 80% of all cases.^1^ The estimated annual incidence of glioma is in the range of 6 out of 100,000 worldwide.^2^ Although relatively rare compared to other malignant tumors, glioblastoma, the most common and deadliest form of glioma, results in a remarkably high mortality rate. The median overall survival is only approximately 19 months regardless of care.^3^ The World Health Organization (WHO) categorizes glioma into 4 subtypes—grades I to IV based on their aggressiveness.^4^ Clinically, gliomas are normally grouped into low-grade glioma (LGG) and high-grade glioma (HGG).

Accurate categorization of LGG and HGG is indispensable to determining the treatment option and the prognosis of patients. Histopathological characterization following biopsy is a routine procedure to diagnose and grade glioma in clinical practice. However, the procedure is expertise-demanding, workforce-intensive, and time-consuming.^5^ To fill in this gap, state-of-the-art medical imaging techniques, especially magnetic resonance imaging (MRI), are widely applied to identify and classify glioma non-invasively, yet both inter- and intraoperator variability cannot be fully avoided. The interpretation of medical images is also highly dependent on the experience and skills of clinicians.

To overcome the aforementioned drawbacks, deep learning (DL), a subset of artificial intelligence (AI), has shown great promise in the automatic classification of medical images.^6^^,^^7^ For instance, the recent advancement of DL algorithms has rendered Food and Drug Administration (FDA) approves a few diagnosis tools for clinical practice.^8^ In our context, numerous independent studies have investigated the performance of DL in glioma classification worldwide. To date; however, there is no systematic review and meta-analysis to assess the diagnostic performance of DL algorithms in grading glioma. This evidence-based study is expected to contribute to the further implementation of DL-based models in routine clinical practice.

## Results

### Study selection and characteristics

**Figure 1:**
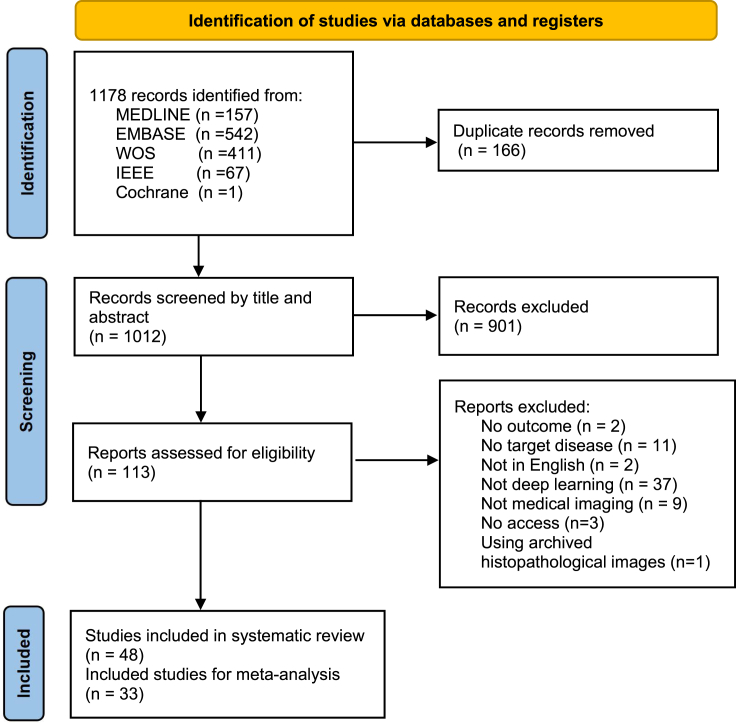
PRISMA flowchart of the study The literature review and record screening processes followed PRISMA (preferred reporting items for systematic reviews and meta-analyses).

Through literature research, we identified 1178 records. After filtering 166 duplicated records, 1012 records stayed. 901 records were excluded further after a title or abstract scanning, followed by filtering 64 records for no outcome, no target disease, no English article, etc. Finally, we included 49 articles that met our inclusion criteria for systematic review, among which 33 articles can fully provide data for meta-analysis (Figure 1).

We totally included 19102 patients. The gold standard was histopathology in all articles. Among the 48 included studies, only 33 studies were included in the meta-analysis due to unextractable or calculation errors in the contingency tables of 15 studies. 52% (17/33) were ≤ 130 sample size, 48% (16/33) were > 130, 21% (7/33) were private data, 79% (26/33) were open data, 27% (9/33) were k-fold cross-validation. 73% (24/33) were random split-sample validation, 73% (24/33) were not using the transfer learning, 27% (9/33) were using, 61% were based on image data (20/33), 39% were based on patient data (13/33), 39% (13/33) used grade Ⅳ to represent HGG, 61% (20/33) used Ⅲ+Ⅳ, 82% (27/33) were based on internal validation, and 18% (6/33) were based on external (Tables 1, 2, and 3).

**Table 1:** Participant demographics for the 48 included studies (33 included in meta-analysis)

First author and year	Participants			
	Inclusion criteria	Exclusion criteria	Number of patients	Mean or median age (SD; range)
Yu et al. (2022)^9^	(1) Histopathologically confirmed and graded glioma according to the current WHO criteria, (2) images acquired before the operation, (3) data sequences including T1w imaging, T2w imaging, FLAIR imaging, and enhanced T1w imaging.	NR	560	NR(NR; NR)
van der Voort et al. (2022)^10^	Newly diagnosed with a glioma and when preoperative pre- and post-contrast T1w, T2w, and T2w-FLAIR scans were available	The absence of one (or more) of the required scans (T1, post-contrast T1, T2w, T2w-FLAIR)	1748	NR(NR; NR)
Danilov et al. (2022)^11^	NR	NR	707	NR(NR; NR)
Chen et al. (2022)^12^	Patients diagnosed with glioma after case diagnostic screening; patients with complete imaging and follow-up data; and patients with complete follow-up records	Patients with other malignant tumors at the same time; patients with other serious underlying diseases or with dysfunction of important organs such as the heart, lung, liver, and kidney; those who died of diseases or accidents other than glioma; and those who suffered from claustrophobia.	66	53.6(11.3; NR)
Tripathi et al. (2022)^13^	The images which contain tumor region	NR	322	NR(NR; NR)
Xiao et al. (2022)^14^	NR	NR	24	NR(NR; NR)
Wang et al. (2022)^15^	NR	NR	378	NR(NR; NR)
Tasci et al. (2022)	NR	NR	369	NR(NR; NR)
Li et al. (2022)^16^	(1) Pathologically diagnosed as diffuse gliomas; (2) high-quality preoperative T1w, T2w, and T1CE MR images were available; (3) age≥18 years; (4) known IDH status (detected by immunohistochemistry or pyrosequencing); and (5) known 1p19q status (detected using fluorescence *in situ* hybridization) for LGGs.	NR	1016	47(NR; NR)
Khazaee et al. (2022)^17^	NR	NR	335	NR(NR; NR)
Jiang et al. (2021)^18^	NR	NR	620	NR(NR; NR)
He et al. (2021)^19^	NR	NR	499	NR(NR; NR)
Haq et al. (2021)^20^	NR	NR	351	NR(NR; NR)
Raghavendra et al. (2021)^21^	NR	NR	461	NR(NR; NR)
Chakrabarty et al. (2021)^22^	NR	NR	2105	57(NR; 47–65)
Yahyaoui et al. (2021)^23^	NR	NR	230	NR(NR; NR)
Yamashiro et al. (2021)^24^	NR	NR	285	NR(NR; NR)
Yao et al. (2021)^25^	(1) All cases accepted MRI scan, diagnosed by clinical imaging physicians and neurosurgeons strictly referring to MRI diagnostic criteria. (2) The patients were diagnosed as BG according to post operative pathological results. (3) The patient had no history of craniocerebral surgery and substantial brain injury. (4) Patients had clear consciousness, were able to communicate normally, and had no mental illness	(1) Cases diagnosed as having cerebral infarction, (2) cases with severe communication disorder or mental illness, (3) cases with intracranial hypertension and other characteristics of intracranial lesions, and (4) patients with liver and kidney dysfunction or allergy to contrast agents	60	55.82(4.18; 20–60)
Bezdan et al. (2021)^26^	NR	NR	NR	NR(NR; NR)
Shen et al. (2021)^27^	(1) Male or female; (2) suspected as malignant glioma on preoperative contrast enhancement MRI; (3) voluntarily signed informed consent of surgical treatment and additional specimen beyond what was needed for routine clinical diagnosis; and (4) no contraindication of ICG.	NR	23	NR(NR; NR)
Irmak et al. (2021),^28^	NR	NR	346	NR(NR; NR)
Al-Saffar et al. (2021)^29^	NR	NR	160	NR(NR; NR)
Hu et al. (2021)^30^	The cases in BraTs had clear MR images and tumor masks, and the cases in TCGA and HuaShan had pathological grading and IDH1 information	NR	800	NR(NR; NR)
Luo et al. (2021)^31^	These cases must contain complete imaging data together with histopathology	NR	655	NR(NR; mostly 18–60)
Decuyper et al. (2021)^32^	A histologically proven glioma of WHO grade II, III or IV, the availability of preoperative T1CE MRI together with a T2 and/or FLAIR sequence of sufficient quality and information on WHO grade, IDH mutation and 1p19q co-deletion status	NR	738	NR(NR; NR)
Gutta et al. (2021)^33^	NR	NR	237	NR(NR; NR)
Ozcan et al. (2021)^34^	NR	NR	104	NR(NR; NR)
Mzoughi et al. (2021)^35^	NR	NR	284	NR(NR; NR)
Koyuncu et al. (2020)^36^	NR	NR	285	NR(NR; NR)
Cinarer et al. (2020)^37^	NR	NR	121	NR(NR; NR)
Mzoughi et al. (2020)^35^^,^^38^	NR	NR	351	NR(NR; NR)
Zhuge et al. (2020)^39^	NR	NR	315	NR(NR; NR)
Naser et al. (2020)^40^	NR	NR	110	46(14; 20–75)
Alis et al. (2020)^41^	Diagnosed with whom grade I to IV according to surgical or biopsy-derived histopathological findings; >18 years of age; having preoperative or pre interventional brain MRI with T2W-FLAIR and contrast-enhanced T1W images	Motion or susceptibility artifacts on MRI; history of radio therapy or chemotherapy for prior brain tumor; residual or recurrent brain tumors; gliomas <1 cm in diameter; incomplete clinical data	181	58(NR; 27–78)
Hollon et al. (2020)^42^	(1) Male or female; (2) subjects undergoing CNS tumor resection at Michigan Medicine, New York Presbyterian/Columbia University Medical Center or the University of Miami Health System; (3) subject or durable power of attorney able to give informed consent; and (4) subjects in whom there was additional specimen beyond what was needed for routine clinical diagnosis.	(1) Poor quality of specimen on visual gross examination due to excessive blood, coagulation artifact, necrosis or ultrasonic damage or (2) specimen classified as out of distribution by the linear discriminant analysis classifier using the Mahalanobis distance-based confidence score.	693	NR(NR; NR)
Sharif et al. (2020)^43^	NR	NR	1211	NR(NR; NR)
Lo et al. (2019)^44^	NR	NR	130	NR(NR; NR)
Gonbadi et al. (2019)^45^	NR	NR	285	NR(NR; NR)
Ali et al. (2019)^46^	NR	NR	285	NR(NR; NR)
Sultan et al. (2019)^28^	NR	NR	73	NR(NR; NR)
Muneer et al. (2019)^47^	NR	NR	20	NR(NR; 30–60)
Anaraki et al. (2019)^48^	NR	NR	688	NR(NR; NR)
Sajjad et al. (2018)^49^^,^^50^	NR	NR	NR	NR(NR; NR)
Shahzadi et al. (2018)^49^	NR	NR	60	NR(NR; NR)
Yang et al. (2018)^51^	NR	NR	113	NR(NR; 10–87)
Al-Zurfi et al. (2018)^52^	NR	NR	30	NR(NR; NR)
Ge et al. (2018)^53^	NR	NR	285	NR(NR; NR)
Khawaldeh et al. (2018)^54^	NR	NR	109	NR(NR; 18–89)
Ye et al. (2017)^55^	NR	NR	274	NR(NR; NR)

SD = standard deviation, WHO = world health organization, T1w = T1 weighted, T2w = T2 weighted, FLAIR = fluid attenuated inversion recovery, NR = not reported, T1CE = T1 contrast-enhanced, MR = magnetic resonance, IDH = Isocitrate dehydrogenase, LGG = low-grade glioma, MRI = magnetic resonance imaging, BG = brain glioma, ICG = indocyanine green, CNS = central nervous system.

**Table 2:** Model training and validation for the 48 included studies(33 included in meta-analysis)

First author and year	Focus	Reference standard	Type of internal validation	External validation	DL versus clinician
Yu et al. (2022)^9^	Brain tumor	Histopathology	Random split-sample validation	No	No
van der Voort et al. (2022)^10^	Brain tumor	Histopathology	Random split-sample validation	Yes	No
Danilov et al. (2022)^11^	Brain tumor	Histopathology	Random split-sample validation	No	No
Chen et al. (2022)^12^	Brain tumor	Histopathology	NR	No	No
Tripathi et al. (2022)^13^	Brain tumor	Histopathology	Random split-sample validation	No	No
Xiao et al. (2022)^14^	Brain tumor	Histopathology	Random split-sample validation	No	No
Wang et al. (2022)^15^	Brain tumor	Histopathology	Random split-sample validation	No	No
Tasci et al. (2022)	Brain tumor	Histopathology	Random split-sample validation	No	No
Li et al. (2022)^16^	Brain tumor	Histopathology	Random split-sample validation	No	No
Khazaee et al. (2022)^17^	Brain tumor	Histopathology	Random split-sample validation	No	No
Jiang et al. (2021)^18^	Brain tumor	Histopathology	Random split-sample validation	No	No
He et al. (2021)^19^	Brain tumor	Histopathology	5-fold cross-validation	Yes	No
Haq et al. (2021)^20^	Brain tumor	Histopathology	Random split-sample validation	No	No
Raghavendra et al. (2021)^21^	Brain tumor	Histopathology	10-fold cross-validation	No	No
Chakrabarty et al. (2021)^22^	Brain tumor	Histopathology	Random split-sample validation	Yes	No
Yahyaoui et al. (2021)^23^	Brain tumor	Histopathology	Random split-sample validation	No	No
Yamashiro et al. (2021)^24^	Brain tumor	Histopathology	Random split-sample validation	No	No
Yao et al. (2021)^25^	Brain tumor	Histopathology	NR	No	No
Bezdan et al. (2021)^26^	Brain tumor	Histopathology	Random split-sample validation	No	No
Shen et al. (2021)^27^	Brain tumor	Histopathology	Random split-sample validation	No	Yes
Irmak et al. (2021)^28^	Brain tumor	Histopathology	5-fold cross-validation	No	No
Al-Saffar et al. (2021)^29^	Brain tumor	Histopathology	Random split-sample validation	No	No
Hu et al. (2021)^30^	Brain tumor	Histopathology	Random split-sample validation	No	No
Luo et al. (2021)^31^	Brain tumor	Histopathology	Random split-sample validation	Yes	No
Decuyper et al. (2021)^32^	Brain tumor	Histopathology	Random split-sample validation	Yes	No
Gutta et al. (2021)^33^	Brain tumor	Histopathology	Random split-sample validation	No	No
Ozcan et al. (2021)^34^	Brain tumor	Histopathology	5-fold cross-validation	No	No
Mzoughi et al. (2021)^35^	Brain tumor	Histopathology	Random split-sample validation	No	No
Koyuncu et al. (2020)^36^	Brain tumor	Histopathology	2-fold cross-validation	No	No
Cinarer et al. (2020)^37^	Brain tumor	Histopathology	Random split-sample validation	No	No
Mzoughi et al. (2020)^38^	Brain tumor	Histopathology	Random split-sample validation	No	No
Zhuge et al. (2020)^39^	Brain tumor	Histopathology	5-fold cross-validation	No	No
Naser et al. (2020)^40^	Brain tumor	Histopathology	5-fold cross-validation	No	No
Alis et al. (2020)^41^	Brain tumor	Histopathology	10-fold cross-validation	No	No
Hollon et al. (2020)^42^	Brain tumor	Histopathology	Random split-sample validation	Yes	Yes
Sharif et al. (2020)^43^	Brain tumor	Histopathology	Random split-sample validation	No	No
Lo et al. (2019)^44^	Brain tumor	Histopathology	10-fold cross-validation	No	No
Gonbadi et al. (2019)^45^	Brain tumor	Histopathology	Random split-sample validation	No	No
Ali et al. (2019)^46^	Brain tumor	Histopathology	Random split-sample validation	No	No
Sultan et al. (2019)^28^	Brain tumor	Histopathology	Random split-sample validation	No	No
Muneer et al. (2019)^47^	Brain tumor	Histopathology	Random split-sample validation	No	No
Anaraki et al. (2019)^48^	Brain tumor	Histopathology	Random split-sample validation	No	No
Sajjad et al. (2018)^50^	Brain tumor	Histopathology	Random split-sample validation	No	No
Shahzadi et al. (2018)^49^	Brain tumor	Histopathology	Random split-sample validation	No	No
Yang et al. (2018)^51^	Brain tumor	Histopathology	5-fold cross-validation	No	No
Al-Zurfi et al. (2018)^52^	Brain tumor	Histopathology	Leave-one-out cross-validation	No	No
Ge et al. (2018)^53^	Brain tumor	Histopathology	Random split-sample validation	No	No
Khawaldeh et al. (2018)^54^	Brain tumor	Histopathology	Random split-sample validation	No	No
Ye et al. (2017)^55^	Brain tumor	Histopathology	Random split-sample validation	No	No

DLdeep learning, NR = not reported.

**Table 3:** Indicator, algorithm, and data source for the 48 included studies(33 included in meta-analysis)

First author and year	Indicator definition			Algorithm		Data source				
	Device	Exclusion of poor-quality imaging	Heatmap provided	Algorithm architecture	Transfer learning applied	Source of data	Number of training/internal/external		Data range	Open access data
							Images	Cases		
Yu et al. (2022),^9^	MRI	Yes	No	3D U-Net	No	Retrospective study, data from BraTS 2019 and the PACS system of Henan Provincial People’s Hospital	NR/NR/NR	448/112/NR	2012–2020	Yes
van der Voort et al. (2022),^10^	MRI	NR	No	CNN	No	Retrospective study, data rom 4 in-house datasets and 5 publicly available datasets	6032/NR/960	1508/NR/240	NR	Yes
Danilov et al. (2022),^11^	MRI	NR	No	DenseNet, Resnest200e	No	Retrospective study, data from N.N. Burdenko Neurosurgery Center, Russia	15957/1773/NR	636/71/NR	2009–2018	No
Chen et al. (2022)^12^	MRI	NR	Yes	CNN	No	Prospective study, data from The First People’s Hospital of Lianyungang	NR/NR/NR	NR/NR/NR	2019.03–2020.03	No
Tripathi et al. (2022),^13^	MRI	Yes	No	Residual networks	Yes	Retrospective study, data from TCIA	6653/739/NR	NR/NR/NR	NR	Yes
Xiao et al. (2022)^14^	Near-infrared fluorescence imaging	NR	Yes	DLS-DARTS	Yes	Prospective study, data from Beijing Tiantan Hospital, Capital Medical University	952//NR	N163R/NR/NR	NR	No
Wang et al. (2022)^15^	MRI	NR	No	3D CNN	No	Retrospective study, data from MICCAI 2020 CPM-Radpath Challenge	NR/NR/NR	305/73/NR	NR	Yes
Tasci et al. (2022)	MRI	NR	No	Xception, IncResNetv2, EfficientNet	Yes	Retrospective study, data from BraTS 2020	17830/4457/NR	NR/NR/NR	NR	Yes
Li et al. (2022),^16^	MRI	Yes	No	2.5D DCNN	No	Retrospective study, data from Beijing Tiantan Hospital	NR/NR/NR	780/236/NR	2014.09–2018.04	No
Khazaee et al. (2022)^17^	MRI	NR	No	EfficientNetB0	No	Retrospective study, data from BraTS 2019	21523/5381/NR	NR/NR/NR	NR	Yes
Jiang et al. (2021)^18^	MRI	Yes	No	SE-ResNeXt	Yes	Retrospective study, data from BraTS 2017 and 2019	21700/9300/NR	NR/NR/NR	NR	Yes
He et al. (2021),^19^	MRI	NR	No	HOMIF	No	Retrospective study, data from TCIA and BraTS 2017	NR/NR/NR	172/42/166 and 228/57/95	NR	Yes
Haq et al. (2021),^20^	MRI	Yes	No	GoogleNet	No	Retrospective study, data from BraTS 2018	20⅛4/NR	NR/NR/NR	NR	Yes
Raghavendra et al. (2021),^21^	MRI	NR	No	VGG-16	No	Retrospective study, data from TCIA	1600/800/NR	NR/NR/NR	NR	Yes
Chakrabarty et al. (2021),^22^	MRI	NR	No	3D-CNN	No	Retrospective study, data from Washing University School of Medicine, BraTS 2018, BraTS 2019, TCIA, and TCGA	415/108/348	415/108/348	2001.02–2019.10	Yes
Yahyaoui et al. (2021),^23^	MRI	NR	No	3D-CNN	No	Retrospective study, data from BraTS 2015 and 2019	190/40/NR	NR/NR/NR	NR	Yes
Yamashiro et al. (2021),^24^	MRI	NR	No	3D-CNN	No	Retrospective study, data from BraTS 2018	6602/46/NR	NR/NR/NR	NR	Yes
Yao et al. (2021)^25^	MRI	NR	Yes	VGG-16	Yes	Prospective study, data from Hunan Cancer Hospital	NR/NR/NR	NR/NR/NR	2019.07–2020.02	No
Bezdan et al. (2021)^26^	MRI	NR	No	CNN-HEHO	Yes	Retrospective study, data from three datasets in TCIA	7200/800/NR	NR/NR/NR	NR	Yes
Shen et al. (2021),^27^	Fluorescent imaging	Yes	Yes	DCNN	Yes	Prospective study, data from Beijing Tiantan Hospital, Capital Medical University	636/296/NR	NR/NR/NR	2019.03–2020.4	No
Irmak et al. (2021),^28^	MRI	NR	No	CNN	No	Retrospective study, data from TCIA	3656/914/NR	NR/NR/NR	NR	Yes
Al-Saffar et al. (2021)^29^	MRI	NR	No	MLP&SVM	No	Retrospective study, data from TCIA	NR/NR/NR	NR/NR/NR	NR	Yes
Hu et al. (2021)^30^	MRI	NR	No	3D U-Net	No	Retrospective study, data from BraTS 2017, TCGA, and HuaShan Hospital	NR/NR/NR	533/267/NR	2001–2018	Yes
Luo et al. (2021),^31^	MRI	NR	Yes	3D U-Net	No	Retrospective study, data from two hospitals including Huashan Hospital and Shanghai International Medical Center	NR/NR/NR	188/411/56	2010–2017	No
Decuyper et al. (2021),^33^	MRI	Yes	No	3D U-Net	No	Retrospective study, data from TCGA, BraTS 2019, and from Ghent University Hospital	NR/NR/NR	528/100/110	NR	Yes
Gutta et al. (2021),^34^	MRI	Yes	No	CNN	No	Retrospective study, data from the Keck Medical Center of the University of the Southern California	560/100/NR	NR/NR/NR	2007.05–2019.01	No
Ozcan et al. (2021)^35^	MRI	NR	No	CNN, AlexNet, GoogLeNet, SqueezeNet	Yes	Retrospective study, data from Amasya University	NR/NR/NR	83/21/NR	2016.12–2019.10	Yes
Mzoughi et al. (2021)^35^	MRI	Yes	No	3D-CNN	No	Retrospective study, data from BraTS 2018	NR/NR/NR	227/57/NR	NR	No
Koyuncu et al. (2020),^37^	MRI	NR	No	GM-CPSO-NN	No	Retrospective study, data from BraTS 2017	NR/NR/NR	143/142/NR	NR	Yes
Cinarer et al. (2020),^38^	MRI	NR	Yes	CNN	No	Retrospective study, data from TCIA	NR/NR/NR	95/26/NR	NR	Yes
Mzoughi et al. (2020)^38^	MRI	Yes	No	3D-CNN	No	Retrospective study, data from BraTS 2018	NR/NR/NR	284/67/NR	NR	Yes
Zhuge et al. (2020),^40^	MRI	Yes	No	2D R-CNN, 3DConvNet	No	Retrospective study, data from BraTS 2018 data and TCIA	NR/NR/NR	252/63/NR	NR	Yes
Naser et al. (2020),^41^	MRI	Yes	No	VGG-16	Yes	Retrospective study, data is available at TCIA	652/163/NR	86/22/NR	NR	Yes
Alis et al. (2020),^42^	MRI	Yes	No	MLP	No	Retrospective study, Istanbul Mehmet Akif Ersoy Thoracic and Cardiovascular Surgery Training and Research Hospital, Turkey	NR/NR/NR	121/60/NR	2013.01–2019.01	No
Hollon et al. (2020),^43^	Stimulated Raman histology imaging	Yes	Yes	Inception-ResNet-v2	No	Prospective study, data from University of Michigan, Columbia University, and University of Miami	NR/NR/NR	NR/NR/74	2015.06–2019.02	No
Sharif et al. (2020)^43^	MRI	NR	No	Inception V3	No	Retrospective study, data from BraTS 2013, 2015, 2017 and 2018	NR/NR/NR	708/428/NR	NR	Yes
Lo et al. (2019),^45^	MRI	NR	No	AlexNet	Yes	Retrospective study, data from TCIA	117/13/NR	NR/NR/NR	NR	Yes
Gonbadi et al. (2019),^46^	MRI	NR	No	CNN	No	Retrospective study, data from BraTS 2017	20⅝0/NR	NR/NR/NR	NR	Yes
Ali et al. (2019),^47^	MRI	NR	No	DCGAN	No	Retrospective study, data from BraTS 2017	5220/1305/NR	NR/NR/NR	NR	Yes
Sultan et al. (2019),^28^	MRI	Yes	No	CNN	No	Retrospective study, data from the Repository of Molecular Brain Neoplasia Data, TCIA	439/77/NR	NR/NR/NR	NR	Yes
Muneer et al. (2019),^48^	MRI	Yes	No	VGG-19	Yes	Retrospective study, data from Government Medical College, India	389/168/NR and 553/228/NR	NR/NR/NR	NR	Yes
Anaraki et al. (2019),^50^	MRI	Yes	No	CNN+GA	Yes	Retrospective study, data from TCIA and Hazrat-e Rasool General Hospital at Tehran, Iran	6500/1500/NR	NR/NR/NR	NR	Yes
Sajjad et al. (2018),^49^	MRI	Yes	No	VGG-19	Yes	Retrospective study, data from Radiopaedia	8⅓0/NR and 2722/908/NR	NR/NR/NR	NR	Yes
Shahzadi et al. (2018)^49^	MRI	NR	No	CNN-LSTM	Yes	Retrospective study, data from BraTS 2015	NR/NR/NR	48/12/NR	NR	Yes
Yang et al. (2018)^51^	MRI	NR	No	AlexaNet and GoogLeNet	Yes	Retrospective study, data from Tangdu Hospital of the Fourth Military Medical College	694/173/NR	90/23/NR	NR	No
Al-Zurfi et al. (2018)^52^	MRI	NR	Yes	DINN	No	Retrospective study, data from TCIA	NR/NR/NR	29/1/NR	NR	Yes
Ge et al. (2018),^54^	MRI	NR	No	Multistream CNN fusion network	No	Retrospective study, data from BraTS 2017 competition	864/216/NR	NR/NR/NR	NR	Yes
Khawaldeh et al. (2018),^55^	MRI	NR	No	AlexNet	No	Retrospective study, data from TCIA	2627/448/NR	NR/NR/NR	NR	Yes
Ye et al. (2017),^56^	MRI	NR	No	3D CNN with GMU fusion	Yes	Retrospective study, data from BraTS 2015	NR/NR/NR	24⅓3/NR	NR	Yes

MRI = magnetic resonance imaging, BraTS = brain tumor segmentation, PACS = picture archiving and communication system, NR = not reported, CNN = convolutional neural network, TCIA = the cancer imaging archive, DLS-DARTS = double-learnable-stem differentiable architecture search, MICCAI = medical image computing and computer assisted interventions, CPM-Radpath = computational precision medicine: radiology-pathology, DCNN = deep convolutional neural network, HOMIF = hierarchical-order multimodal interaction fusion network, VGG = visual geometry group network, TCGA = the cancer genome atlas, HEHO = hybridized elephant herding optimization, MLP&SVM = multi-layer perceptron and support vector machine, GM-CPSO-NN = Gauss-map-based chaotic particle-swarm optimization neural network, R-CNN = residual convolutional neural network, DCGAN = deep convolutional generative adversarial networks, GA = genetic algorithms, LSTM = long short term memory, DINN = deep iteration matrix of neural network, GMU = gated multimodal unit.

### Pooled performance of DL algorithms

**Figure 2:**
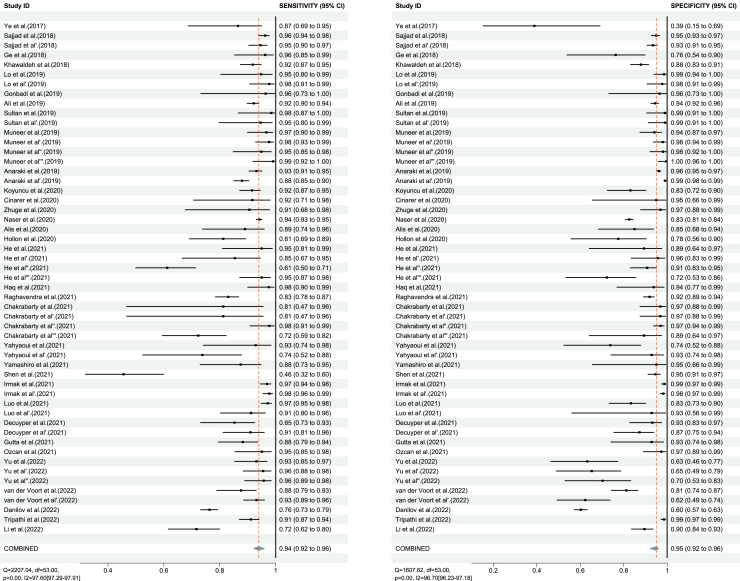
Forest plot of the pooled performance of deep learning (DL) algorithms, based on all 54 tables in 33 studies.

Among 33 articles with sufficient data, when considering all the records in line with our criteria (including all results in every study), the overall (54 contingency tables) pooled sensitivity (SE) and specificity (SP) were 94% (95% CI: 91–95%) and 93% (95% CI: 91–95%) (Figure 2), with the area under the curve (AUC) of 0.98 (95% CI: 0.96–0.99) for all DL algorithms (Figure 4A).

**Figure 3:**
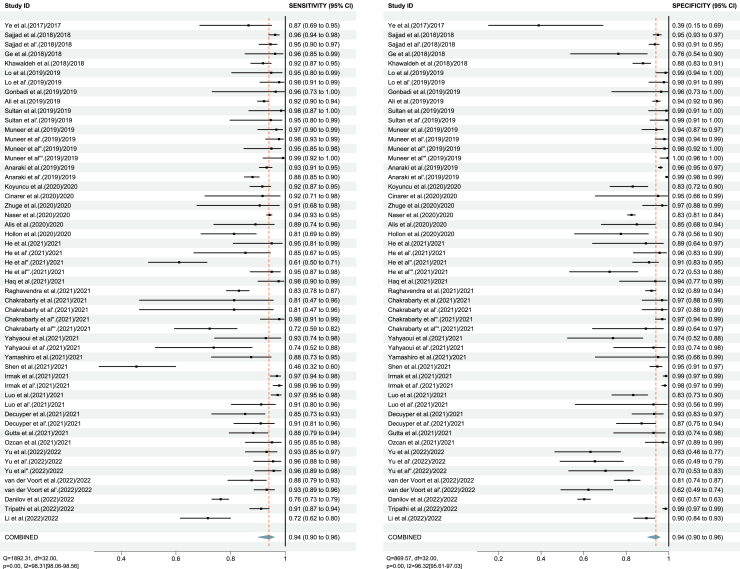
Forest plot of the pooled performance of deep learning (DL) algorithms, reporting the highest accuracy of 33 studies.

**Figure 4:**
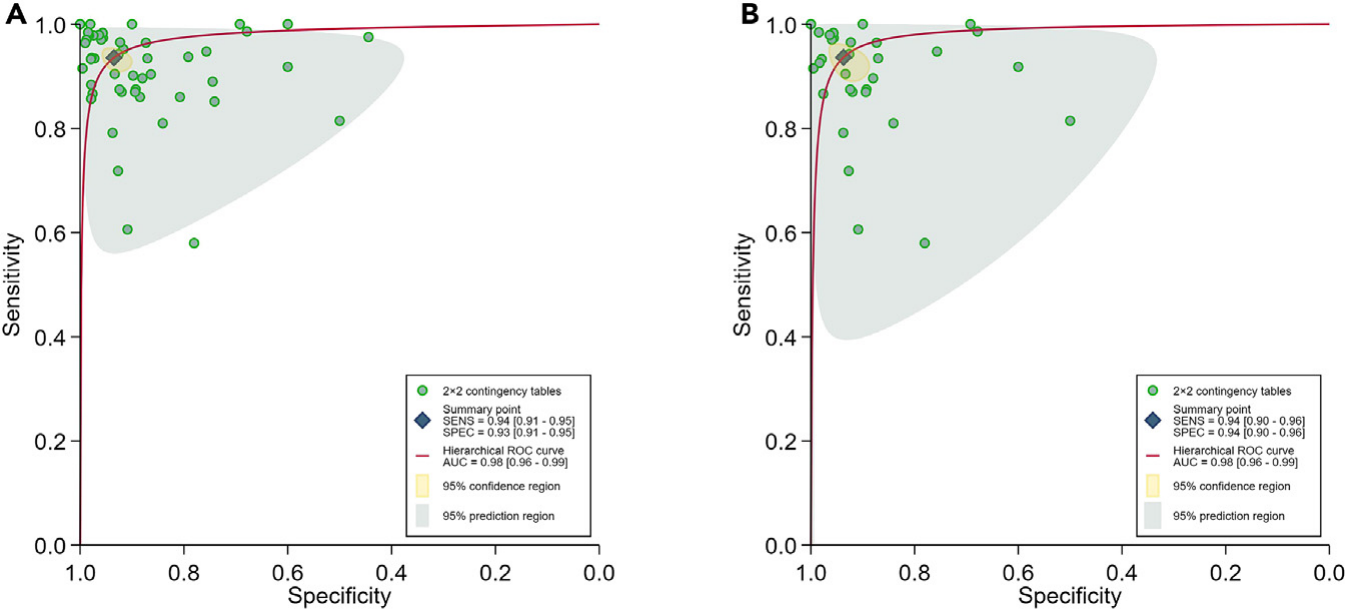
Overall pooled performance of deep learning (DL) algorithms on glioma grading (A) Hierarchical summary receiver operating characteristic (HSROC) curve of all contingency tables (54 tables). (B) HSROC curve reporting the highest accuracy (33 tables).

Considering the problem of reusing samples, we also used the highest accuracy as the criteria to select only one reported performance for each study. The pooled results of highest accuracy for SE and SP were 94% (95% CI: 90–96%) and 94% (95% CI: 90–96%) (Figure 3), with the AUC of 0.98 (95% CI: 0.96–0.99) (Figure 4B).

### Heterogeneity analysis

The overall (54 contingency tables) pooled analysis showed I^2^ = 97.6% in SE and I^2^ = 96.7% in SP. Besides, the highest accuracy pooled analysis indicated I^2^ = 98.31% and 96.32% in SE and SP, respectively.

To explore the causes of heterogeneity, we applied meta-regression containing suspectable variables. Including:1) sample size; 2) data sharing; 3) type of internal validation; 4) transfer learning applied; 5) data unit; 6) classification; and 7) type of validation. Among the first 5 variables, data sharing showed no statistical significance (p = 0.39 in SE, p = 0.91 in SP), but the rest 4 variables showed significance at least in one of SE or SP, which indicated heterogeneity. As for the classification and type of validation, both of them had statistical significance in SE and SP, revealing heterogeneity (Table S1).

### Subgroup analysis

All variables included in the meta-regression were divided into 2 groups for subgroup analysis.

Sample size: In ≤ 130 subgroup, the SE was 96% (95% CI: 92–98%), SP was 92% (95% CI:85–96%) and AUC was 0.98 (95% CI: 0.97–0.99), while in > 130 subgroup, they were 91% (95% CI: 85–95%), 94% (95% CI: 90–97%), and 0.98 (95% CI: 0.96–0.99), respectively. Heterogeneity still existed in two subgroups (≤ 130: I^2^ = 74.08% in SE and 80.42% in SP; > 130: I^2^ = 99.05% in SE and 97.73% in SP) (Figures S1 and S18).

Data sharing: In private data subgroup, the SE was 88% (95% CI: 73–95%), SP was 82% (95% CI:74–88%) and AUC was 0.89 (95% CI: 0.86–0.92), while in open data subgroup, they were 95% (95% CI: 92–97%), 96% (95% CI: 92–97%), and 0.99 (95% CI: 0.97–0.99), respectively. Heterogeneity still existed in two subgroups (private data: I^2^ = 98.71% in SE and 96.84% in SP; open data: I^2^ = 94.31% in SE and 91.89% in SP) (Figures S2 and S9).

Type of internal validation: In k-fold cross-validation subgroup, the SE was 96% (95% CI: 90–99%), SP was 97% (95% CI:89–99%), and AUC was 0.99 (95% CI: 0.98–1.00), while in random split-sample validation subgroup, they were 92% (95% CI: 88–95%), 92% (95% CI: 88–95%), and 0.97 (95% CI: 0.95–0.98), respectively. Heterogeneity still existed in two subgroups (k-fold cross-validation: I^2^ = 98.50% in SE and 97.92% in SP; random split-sample validation: I^2^ = 98.27% in SE and 95.92% in SP) (Figures S3 and S10).

Transfer learning applied: In no applied subgroup, the SE was 94% (95% CI: 90–97%), SP was 93% (95% CI:88–96%), and AUC was 0.98 (95% CI: 0.96–0.99), while in applied subgroup, they were 93% (95% CI: 85–97%), 95% (95% CI: 93–97%), and 0.98 (95% CI: 0.96–0.99), respectively. Heterogeneity still existed in two subgroups (no applied: I^2^ = 98.70% in SE and 97.12% in SP; applied: I^2^ = 95.56% in SE and 90.05% in SP) (Figures S4 and S11).

Data unit: In image subgroup, the SE was 95% (95% CI: 90–97%), SP was 96% (95% CI:92–98%), and AUC was 0.99 (95% CI: 0.97–0.99), while in patient subgroup, they were 92% (95% CI: 87–95%), 88% (95% CI: 81–92%), and 0.96 (95% CI: 0.94–0.97), respectively. Heterogeneity still existed in two subgroups (image: I^2^ = 99.21% in SE and 98.21% in SP; patient: I^2^ = 86.03% in SE and 78.39% in SP) (Figures S5 and S12).

Classification: In grade Ⅳ represented HGG subgroup, the SE was 94% (95% CI: 91–96%), SP was 93% (95% CI:89–96%) and AUC was 0.98 (95% CI: 0.96–0.99), while in grade Ⅲ+Ⅳ represented HGG subgroup, they were 93% (95% CI: 89–96%), 94% (95% CI: 89–96%) and 0.98 (95% CI: 0.96–0.99), respectively. Heterogeneity still existed in two subgroups (Ⅳ: I^2^ = 85.92% in SE and 92.73% in SP; Ⅲ+Ⅳ: I^2^ = 98.60% in SE and 97.41% in SP) (Figures S6 and S13).

Type of validation: In internal subgroup, the SE was 94% (95% CI: 91–96%), SP was 94% (95% CI:92–96%) and AUC was 0.98 (95% CI: 0.97–0.99), while in external subgroup, they were 92% (95% CI: 88–95%), 82% (95% CI: 58–94%) and 0.94 (95% CI: 0.91–0.96), respectively. Heterogeneity still existed in two subgroups (internal: I^2^ = 97.83% in SE and 95.78% in SP; external: I^2^ = 48.29% in SE and 93.30% in SP) (Figures S7 and S14).

### Publication bias evaluation

In the overall pooled analysis, the p value of Deeks’ funnel plot was 0.873. In the highest accuracy pooled analysis, which value was 0.493. Neither of these analyses indicated publication bias (Figure S15).

### Quality assessment

The quality of the total 48 included studies was assessed using QUADAS-2 and a summary of the risk of bias and applicability concerns for 48 studies was provided in Figure S16. The detailed results were also supplied in the Figure S17. In the patient selection domain of risk of bias, 35 studies were deemed high or unclear risk due to unreported inclusion and exclusion criteria, or unknown patient enrollment procedure. For index test, 35 studies were considered at an unclear risk because of a lack of pre-specified thresholds. No risk of bias was observed in the reference standard domain while the bias of flow and timing was unclear for 9 studies due to the following exclusion of patients for further analysis.

In the applicability concerns, 25 studies were considered at high or unclear applicability in the patient selection domain, 11 studies at unclear applicability in the index test domain, while low applicability concerns were observed for all studies in the reference standard domain.

Table 4 introduced supplemental figures, tables, and other information.

**Table 4:** introduction of supplementary

Title	Introduction
Data S1	Search strategies for different databases
Figure S1	HSROC curves of different sample sizes
Figure S2	HSROC curves of open access data or not
Figure S3	HSROC curves of different internal validation types
Figure S4	HSROC curves of using transfer learning or not
Figure S5	HSROC curves of different data units
Figure S6	HSROC curves of glioma classification types
Figure S7	HSROC curves of validation types
FigureS8	Forest plot of different sample sizes
Figure S9	Forest plot of open access data or not
Figure S10	Forest plot of internal validation type
Figure S11	Forest plot of using transfer learning or not
Figure S12	Forest plot of data unit
Figure S13	Forest plot of glioma classification types
Figure S14	Forest plot of validation types
Figure S15	Funnel plot
Figure S16	QUADAS-2 summary plot
Figure S17	QUADAS-2 plot for each detailed item
Table S1	Meta regression result

## Discussion

Up to now, previous systematic reviews and meta-analyses on AI applied to glioma focused on the following topics: prediction of AI on the molecular classification of glioma,^57^^,^^58^^,^^59^ prediction the prognosis,^60^ differential diagnosis between glioma and other brain tumors,^61^^,^^62^ glioma image segmentation,^63^ and grading of glioma.^64^^,^^65^^,^^66^ As for the grading of glioma, two studies pointed out the current obstacles of AI deployment,^64^^,^^65^ and one study conducted a meta-analysis on machine learning (ML) of grading.^66^ However, though DL showed sufficient superiority in other cancers, such as cervical cancer and breast cancer,^67^ it still remained vacant in grading glioma. Moreover, it is notable that glioma grading combined with AI exhibits some traits that are not present in other cancers, such as extensive use of public databases, and the importance of classification for prognosis.^22^^,^^66^ Glioma grading based on open databases accounted for 26 of 33 in our study, which indicated a large number of applications for open-access data. Besides, tumor grading is critical in glioma progression and prognosis; glioblastoma multiforme (grade IV) has a 5-year survival rate of less than 5%^68^ while the survival rate of 15-year for grade II glioma is 86%.^68^^,^^69^ Compared with traditional diagnostic methods, DL has advantages such as shorter diagnostic time, labor saving, and the ability to improve cancer screening in low-resource areas.^68^ Thus, DL performance on glioma grading is worth lots of attention.

In our study the SE and SP were 94% (95% CI: 90–96%) and 94% (95% CI: 90–96%), respectively. A pertinent systematic review and meta-analysis focused on ML, which pooled 5 studies and showed the pooled SE and SP were 96% (95% CI: 93–98%) and 90% (95% CI: 85–94%).^66^ From above results we can’t differentiate the superiority of DL over ML. The potential explanation is that DL outperforms ML when the sample size is huge.^70^ However, in our study, the median sample size was 130, which indicated that most of the eligible studies belonged to small sample data. Thus, DL performance might be hindered by data size limitations. Moreover, DL automatically extracts image features while ML mainly relies on images whose features have been extracted before, usually by clinicians or other experts.^64^ This trait of DL makes it strongly hinge on the quality of images. In our study, only 16 of 33 studies excluded poor-quality images before processing. However, since DL algorithm after exclusion of poor-quality images will hardly present the real clinical setting; therefore, DL models should limit the exclusion of images.

It was noteworthy that we assessed DL from two different criteria: one used all available contingency tables; the other used only one contingency table reporting the highest accuracy from each article. The pooled results (SE:94% (95% CI: 91–95%), SP:93% (95% CI: 91–95%), and AUC:0.98 (95% CI: 0.96–0.99)) were modest worse than those in highest accuracy (SE:94% (95% CI: 90–96%), SP: 94% (95% CI: 90–96%), and AUC: 0.98 (95% CI: 0.96–0.99)). Besides, when the sample size increased, the confidence interval narrowed, which explained the phenomenon that CIs of overall datasets was narrower than the highest accuracy datasets. By the repeating use of samples in the overall analysis, it factitiously added the sample volume of duplicated articles. The phenomenon of single article containing multiple DL algorithms is commonplace in the oncology field,^9^^,^^22^^,^^71^ which requests further meta-analysis of diagnostic evaluations to be equipped with the method to merge multiple sets within each study. Such an approach has already been used in clinical trials, but still remains vacant in diagnostic trials.^72^

As for subgroup analysis, in sample size (≤ 130 or > 130) results, we didn’t find the expected results that the bigger sample size group performed better than a smaller one. In views of the forest plot and original data, we could see that the > 130 sample size group contained narrower confidence intervals than the ≤ 130 group, but still incorporated poor results such as Shen et al. with 296 images (only 60.6% SE),^27^ and Danilov et al. with 1773 images (only 58% SE and 78% SP).^11^ Thus, the heterogeneity was still high in the > 130 sample size group, whereas it was decreased in the ≤ 130 group (≤ 130: I^2^ = 74.08% in SE and 80.42% in SP; > 130: I^2^ = 99.05% in SE and 97.73% in SP). Our study implied that data quality varied enormously in the glioma classification area, which inevitably hindered us from drawing the conclusion of DL. Also, further study should embrace big data, which are the exact field DL experts in.^73^

In data sharing (open data/private data) subgroup analysis, we found that DL in open data performed superior than private data (SE: 95% vs. 88%; SP: 96% vs. 82%; AUC: 0.99 vs. 0.89). Glioma open-access databases, such as The Cancer Imaging Archive (TCIA)^74^ and Brats^75^ were used in 37 out of 49 studies included in the systematic review. Besides, these open databases were the standard databases in MICCAI (an AI competition held by Medical Image Computing and Computer Assisted Intervention Society), which is the top academic competition and play a cardinal role in AI. In these databases, the images were labeled and quality-checked by experienced clinical specialists and had been processed with standardization. By contrast to private data, the images of open data were of higher quality, which led to better results in the subgroup. Here, our results once again emphasized the great importance of data preprocessing. Besides, some recent efforts, which devoted toward standardization of preprocessing, preprocess datasets the same way as it is done for Brats. Thus, the data processed from these tools can be used alongside Brats data.^76^ In this advanced field, there are not any regulations to ensure uniformity and high quality of preprocessing. Recently, the US Food & Drugs Administration (FDA) has approved serial available AI/ML-based medical devices and algorithms to standardize the process of AI tool development, which means that developers of algorithms go through rigorous evaluation before they launch their program.^8^

As for the type of internal validation, k-fold cross-validation outperformed random split-sample validation (SE: 96% vs. 92%; SP: 97% vs. 92%; AUC 0.99 vs. 0.97). K-fold cross-validation fits in small samples data and can conduct parameter tuning through multiple times of training and testing sets segmentation in the same database.^77^ Therefore, it can improve the efficiency of data utilization. However, random split-sample validation only carries out cross-validation through one training set and test set segmentation, which has a large uncertainty, hardly to achieve true randomization.^63^ In our study, since only 27% of the included research used k-fold cross-validation, we appeal for more k-fold cross-validation to be used in this field in future.

Another DL-related item was transfer learning, but in this study, we couldn’t tell the superiority of transfer learning. Transfer learning enables a previously trained model used in another domain. Therefore, it skips the effort required to collect training data.^78^ An article indicated that due to differences in demographic characteristics, transfer learning used on underrepresented patients might exert a negative influence on AI integration with oncology.^79^ In this study, except for transfer learning used for open data, there were many studies using it from open data on private data with the discrepancy in the patient characteristics with previous studies. For example, Shen et al., based on patients from a Chinese hospital, transferred the DL method from another study using the Brats database containing patients of the USA,^27^ which indicated poor SE of 60.6%. Therefore, our result doesn’t mean that transfer learning isn’t suitable in this domain, since transfer learning from different population might create dissatisfying results due to populations but not the AI algorithm itself. Further, we hope that in the future when researchers apply transfer learning, they are supposed to take population heterogeneity into consideration.

With suspect to using images or patients as data unit, we concluded that image-based dataset showed better SE (95% vs. 92%) and SP (96% vs. 88%). It is noteworthy that whether the study reported image number or patient number, the AI process is still based on the image. Therefore, articles only reporting patient number were somehow without preciseness. Especially in contingency tables, if the article only provided patient numbers instead of image numbers, we inevitably underestimated the sample size of these studies, since every patient usually generates more than 1 image. Therefore, to obtain high-quality results, articles should exactly report not only patient numbers but also image numbers, and better report the image-contained results in contingency tables.

In the classification of HGG and LGG, 56% contingency tables (30/54) included in our study defined Ⅳ grade as HGG, such as Luo et al.^31^ and Decuyper et al.,^32^ while others defined Ⅲ and Ⅳ(24/54) grades as HGG, such as Danilov et al.^11^ and Li et al.^16^ Here, our study found that Ⅳ represented HGG in classification was similar to Ⅲ+Ⅳ(SE: 94% vs. 93%; SP:93% vs. 94%; AUC 0.98 vs. 0.98). In WHO glioma classification, diffuse glioma is defined as WHO grade II, anaplastic, or in case of 1p/19q-non-codeleted tumor as grade III and glioblastoma as grade IV.^80^ In the image diagnosis, glioblastoma has the most invasive feature, which can be distinguished from diffuse astrocytoma and anaplastic astrocytoma.^81^ However, distinguishing anaplastic astrocytoma and diffuse astrocytoma features in the images is another story. If researchers deem Ⅲ+Ⅳ as HGG, which is to distinguish grade Ⅲ from grade Ⅱ, it cannot be easy to achieve.^82^ Recent studies indicated that molecular profiling differences existing between these two grades might be used in classification. Important molecular diagnostic markers, such as isocitrate dehydrogenase (IDH) mutation,^83^ 1p/19q co-deletion^84^ and O-6-methylguanine-DNA methyltransferase promoter methylation,^85^ had been included into guideline since WHO glioma classification 2016.^80^ Therefore, in the future, DL algorithms evaluation in image-based glioma grading should also take molecular diagnostic markers into consideration, especially in the distinguishment of grade Ⅱand Ⅲ glioma.

As for internal validation or external validation, in our study, internal subgroup was superior to external subgroup (SE: 94% vs. 92%; SP: 94% vs. 82%; AUC 0.98 vs. 0.94). Internal validation is that in the validation phase, the testing set is separated from the original dataset, whereas external validation is that using a completely independent dataset out of the original one.^86^ Though DL performed inferiorly in the external group, we still appeal to further studies to apply external instead of internal validation. One of the major limitations of including studies is that the majority of them didn’t implement external validation, which made them hard to be generalized and reproduced. DL development is supposed to consider data extrapolation. DL algorithm should generalize to the real-world usage, which means not only exerts well in online database but also can show acceptable quality in clinical practice, such as being auxiliary with hospital clinician or commune healthcare worker. Xian et al. used DL in near-infrared fluorescence imaging to help intraoperative diagnosis,^86^ which requested not only accuracy but also celerity. Besides, as for the application of AI technology in low-resource areas, the acceptance ability of healthcare workers also needs to be considered.^87^ Therefore, in order to facilitate the practical application and promotion of DL merging with glioma classification, in addition to algorithm optimization, time of DL diagnosis, maneuverability, protection of patient information, etc, should also be under rigorous design.

To improve DL algorithms combined with glioma, based on previous analysis of our research, we try to summarize limitations in this field: 1) diagnostic trials require standard method for data merging for AI; 2) small sample size; 3) poor-quality image preprocessing; 4) not standard algorithm development; 5) not standard data report; 6) different definition of HGG and LGG; and 7) poor extrapolation.

Moreover, we offered the following suggestions for further separate studies in AI development in glioma: 1) use open databases; 2) before disclosure, be approved by FDA or other authoritative institutions first; 3) embrace big data; 4) encourage the use of k-fold cross-validation; 5) consider the consistency of characteristics of the two studies populations when using transfer learning; 6) report the number of images in contingency tables; and 7) encourage external validation. Besides, we expect diagnostic trials to provide normative guidelines for data fusion, and top institutes can convene specialists from regarding professions, such as clinicians, AI engineers, pathologists to standardize image preprocessing, and AI development.

Our study used meta-analysis to integrate articles about the performance of DL algorithms in image-based glioma grading. To the best of our knowledge, this is the first meta-analysis to explore the performance of DL in this field. When analyzing the full data, we considered both the full use of data and the selection of representative data (the highest accuracy) of an article, which might be a reference way in the absence of the standard of combining multiple sets of data in diagnostic tests. We further used meta-regression to explore the source of heterogeneity, which indicated that sample size, data sharing, type of internal validation, transfer learning applies, classification, and type of validation did play an important role in heterogeneity. In subgroup analysis, we find that DL displayed with distinguishment in different subgroups. In explanation of difference, we gave recommendations under which DL performs more superior. More importantly, we provided suggestions on how DL should be normalized in glioma grading in the future based on the dilemma of DL development that existed in our results.

However, there are still some limitations in this study. Our results showed high heterogeneity, which was not significantly reduced in subgroup analysis. The items used in subgroup analysis were proved to exert an impact on heterogeneity in meta-regression, and they were considered as possible heterogeneity sources in previous studies. Liu et al. conducted a pooled analysis to evaluate the performance of healthcare workers versus DL, which implied the separation of DL from clinician data in studies.^88^ Besides, DL-related items also contained huge diversity, such as performing external validation or internal validation,^89^ using open-access dataset or not,^90^ the application of transfer learning or not, as well as the validation type.^91^ Therefore, items included in this study were scientific and have been shown to contribute to heterogeneity. Moreover, high heterogeneity was common in studies of the convergence of AI and medicine, such as the DL study of breast and cervical cancer,^67^ glioma segmentation,^63^ gastrointestinal cancer classification, and prognostication^92^ and so on. Admittedly, the reason why heterogeneity was not reduced might also be explained by other possible factors, such as prospective or retrospective studies and DL diagnoses or clinician diagnoses.^67^ Due to the scarcity of articles containing prospective studies (⅔3) or with a comparison of DL versus clinician (3/33) in our study, we couldn’t perform meta-regression on them. Another limitation is that in glioma classification, we failed to incorporate molecular information, which is becoming increasingly important, since it marks a more refined classification of patients and is critical for clinical treatment choice and prognosis.^64^ In addition, the QUADAS-2 assessment was not tailored for AI-based studies, which resulted in risk of bias and applicability concerns.

In conclusion, though the SE, SP, and AUC of DL algorithms are high in glioma grading, we still couldn’t prove the superiority of DL over ML. In the whole dataset pooled analysis, we considered both the full use of data and the selection of representative data (the highest accuracy) for each article. Our study suggested that the results were highly heterogeneous and sample size, data sharing, type of internal validation, transfer learning applies, classification, and type of validation were the possible reasons. In subgroup analysis, we didn’t find the bigger sample size group displayed better than the smaller one. DL in open data appeared superior to private data. As for type of internal validation, k-fold cross-validation outperformed random split-sample validation. In transfer learning use, we couldn’t tell the superiority of transfer learning appliance in comparison to not use. Image-based datasets showed better results than the patients-based ones. In the classification of HGG and LGG, our study indicated that Ⅳ represented HGG excelled Ⅲ+Ⅳ. As for internal validation or external validation, in our study, the internal subgroup was superior to the external. Besides, from the perspective of the whole results of our study, we strongly recommend separate research:1) use open databases; 2) before disclosure, be approved by FDA or other authoritative institutions first; 3) embrace big data; 4) encourage the use of random split-sample validation; 5) consider the consistency of characteristics of the two studies populations when using transfer learning; 6) report the number of images in contingency tables; 7) include molecular typing results to assist diagnosis if grade III incorporated in HGG; 8) encourage external validation; and 9) incorporation of AI-based quality of reporting tools (such as Quadas-AI, Probast-AI or Tripod-AI). Moreover, we can’t emphasize more on normalization of image extraction, preprocessing, and algorithm development in this field. However, since the heterogeneity still remained in subgroups, these recommendations should be considered cautiously.

## STAR★Methods

### Key resources table

**Table:** 

REAGENT or RESOURCE	SOURCE	IDENTIFIER
**Deposited data**		

Original data	this paper	https://doi.org/10.57760/sciencedb.07885

**Software and algorithms**		

Original code	this paper	https://doi.org/10.57760/sciencedb.07886

### Resource availability

#### Lead contact

Further information and requests for resources and reagents should be directed to and will be fulfilled by the lead contact, Wanyi Sun (sunwy1998@foxmail.com).

#### Materials availability

This study did not use any materials.

### Method details

#### Search strategy and eligibility criteria

The online database search consisted of Ovid-Medline, Embase, IEEE Xplore, Web of Science Core Collection, and the Cochrane Library for studies continuously published from 1st January 2015 until 16^th^ August 2022. Keywords including ‘glioma OR astrocytoma OR glioblastoma’, ‘DL OR AI NOT traditional ML, ‘diagnosis OR classification OR grading’, and ‘performance OR (sensitivity and specificity) OR area under the curve’ were used to explore pertinent studies. The detailed search strategies among these 5 databases were available in the supplemental information.

Any studies reporting the performance of DL algorithms on grading gliomas were included during the identification phase while only duplicates were removed. Further exclusion criteria were applied during the first round screening phase as follows: (1) studies published before January 1^st^ 2015, when DL algorithms were not mature at this stage^56^; (2) non-clinical studies, reviews, letters, or comments; (3) studies to investigate the glioma segmentation; (4) investigations exclusively on brain tumor classifications and genetic or molecular subtypes of glioma, not related to glioma grading.

Eligibility assessment was then performed by two independent researchers (W.S. and C.S.) who had reviewed the titles and abstracts of all records, during which the full-text studies were reviewed and assessed in detail. Discrepancies were settled by a third senior researcher (P.X.). The studies using archived histopathological images, not in English, not using DL algorithms for classification, reporting no classifying outcomes, with no target disease, and no access were ruled out from the study while the remaining studies were included for systematic review and further meta-analysis.

#### Data extraction

Two independent researchers (W.S. and C.S.) reviewed the full-text articles (and supplemental information if available) and extracted study characteristics (patients’ information, imaging modality, DL algorithms, etc.) and diagnostic performance of DL (true-positives, false-positives, true-negatives, and false-negatives) into a predetermined data extraction form. Conflicts were resolved through a team discussion and consensus. All classifications other than HGG and LGG were then converted into an exclusive binary classification of HGG and LGG to generate contingency tables for meta-analysis. The extracted data was used to calculate the pooled sensitivity, specificity, and area under the curve (AUC).

### Quantification and statistical analysis

To assess the performance of DL algorithms to differentiate HGG from LGG, the definition of true positive (TP) was set for HGG while that of true negative (TN) was LGG. The included studies with inconsistent definition were redefined for our calculation. A hierarchical summary receiver operating characteristic (HSROC) curve with 95% confidence intervals (CI) and 95% prediction regions was employed to assess the overall performance of DL algorithms along with diagnostic parameters including pooled AUC, sensitivity, and specificity.^93^^,^^94^^,^^95^ Given the inherent differences among the included studies, a bivariate random-effect model was implemented. Heterogeneity was estimated using the Higgins inconsistency index (I^2^) statistic, of which 50% was defined as moderate and higher than 75% was defined as high respectively. Important variables affecting heterogeneity were assessed using meta-regression. The variables finally included in meta-regression analysis were: 1) sample size (≤130/>130; 130 is the median of sample size); 2) data sharing (open data/private data); 3) type of internal validation (random split-sample validation/k-fold cross-validation); 4) transfer learning applied (applied/no applied); 5) data unit(image/patient); 6) classification (grade Ⅳ represented HGG/grade Ⅲ+ Ⅳ represented HGG; According to the WHO classification standard and the actual classification of articles); 7) type of validation(internal/external). The first 5 variables did not differ in multiple DL performances in one study, so the meta-regression based on the highest accuracy pooled analysis was applied for these 5 factors. However, the rest 2 variables displayed diversely in one study, which requested the overall pooled analysis. Further subgroup analysis was performed by variables with statistically significant heterogeneity contribution. Meta-analysis was only conducted only when the number of studies was equal to or greater than three. Data analysis was conducted by STATA (version 15.1) software and the MIDAS module was used. The *p* value less than 0.05 was considered statistically significant. The original data and code were deposited at Science Data Bank and were publicly available (key resources table).

#### Quality assessment

The risk of bias and applicability concerns of the included studies were assessed by two researchers (W.S. and C.S.) using the Quality Assessment of Diagnostic Accuracy Studies 2 (QUADAS-2) tool,^96^ which allows for more transparent rating of bias and applicability of diagnostic accuracy studies. QUADAS-2 tool consists of four key domains: patient selection, index test, reference standard, flow and timing. Publication bias was assessed by a funnel plot.

### Additional resources

The study was registered with the open-access PROSPERO International prospective register of systematic reviews (CRD42022360385). The study was performed strictly following the Preferred Reporting Items for Systematic Reviews and Meta-Analyses (PRISMA) 2020 statement.^97^ Both ethical approval and informed consent were not applicable since this study was a secondary analysis of publicly available data.

## Data Availability

• The original database is stored in the Science Data Bank to make publicly accessible. DOI is listed in the key resources table. • The original code is stored in the Science Data Bank to make publicly accessible. DOI is listed in the key resources table. • Any additional information required to reanalyze the data reported in this paper is available from the lead contact upon request.
