# Supplementary material for: Performance of deep learning algorithms to distinguish high-grade glioma from low-grade glioma: A systematic review and meta-analysis
Source: iScience. 2023 May 5;26(6):106815. doi: 10.1016/j.isci.2023.106815 (PMC10209541; doi:10.1016/j.isci.2023.106815)
Supplement: Document S1. Figures S1–S17, Table S1, and Data S1 [file mmc1.pdf]

iScience, Volume *26*

Supplemental information

Performance of deep learning algorithms to distinguish high-grade glioma from low-grade glioma: A systematic review and meta-analysis

Wanyi Sun, Cheng Song, Chao Tang, Chenghao Pan, Peng Xue, Jinhu Fan, and Youlin Qiao

Data S1 Search strategies, related to Table 4.

MEDLINE (via Ovid):
1 ((“*artificial intelligence*” or “*machine learning*” or “*deep learning*” or “*neural network*”) not (decision tree or random forest or nearest neighbo$ or naïve bayes or “*vector machine*”)).ab,hw,kf,ti,nm.
2 diagnosis.ab,hw,kf,ti,nm.
3 screen*.ab,hw,kf,ti,nm.
4 2 or 3
5 performance.ab,hw,kf,ti,nm.
6 sensitivity.ab,hw,kf,ti,nm.
7 specificity.ab,hw,kf,ti,nm.
8 accuracy.ab,hw,kf,ti,nm.
9 area under the curve.ab,hw,kf,ti,nm.
10 auc.ab,hw,kf,ti,nm.
11 “calibrat*” .ab,hw,kf,ti,nm.
12 5 or 6 or 7 or 8 or 9 or 10 or 11
13 “glioma*” .ab,hw,kf,ti,nm.
14 “glioblastoma*” .ab,hw,kf,ti,nm.
15 “astrocytoma*” .ab,hw,kf,ti,nm.
16 13 or 14 or 15
17 4 and 16
18 12 and 17
19 1 and 18

Embase
#1 ‘artificial intelligence’/exp OR ‘machine learning’/exp OR ‘deep learning’/exp OR ‘artificial neural network’
#2 ‘decision tree’/exp OR ‘random forest’/exp OR ‘Bayesian learning’/exp OR ‘support vector machine’/exp
#3 ‘diagnosis’/exp OR ‘diagnostic procedure’/exp OR ‘screening’/exp
#4 ‘performance’/exp OR ‘sensitivity and specificity’/exp OR ‘diagnostic accuracy’/exp OR ‘area under the curve’/exp OR ‘auc’/exp
#5 ‘glioma*’ OR ‘glioblastoma*’ OR ‘astrocytoma*’
#6 #3 AND #5
#7 #4 AND #6
#8 #1 NOT #2
#9 #7 AND #8

IEEE Xplore
((“ALL metadata”:artificial intelligence OR “ALL metadata”:machine learning OR “ALL metadata”:deep learning OR “ALL metadata”:neural network) NOT (“ALL metadata”:decision tree OR “ALL metadata”:naïve bayes OR “ALL metadata”:nearest neighbor OR “ALL metadata”:vector machine)) AND (“ALL metadata”:screen* OR “ALL metadata”:diagnosis) AND (“ALL metadata”:performance OR “ALL metadata”:sensitivity and specificity OR “ALL metadata”:accuracy OR “ALL metadata”:area under the curve) AND (“ALL metadata”:glioma* OR “ALL metadata”:glioblastoma* OR “ALL metadata”:astrocytoma*)

Cochrane library
#1 (artificial intelligence):ti,ab,tw OR (machine learning):ti,ab,kw OR (deep learning):ti,ab,kw OR (neural network, computer): ti,ab,kw
#2 (decision tree):ti,ab,kw OR (random forest):ti,ab,kw OR (Bayesian learning):ti,ab,kw OR (vector machine):ti,ab,kw
#3 (diagnosis):ti,ab,kw OR (screen*):ti,ab,kw
#4 (performance):ti,ab,kw OR (sensitivity and specificity):ti,ab,kw OR (accuracy):ti,ab,kw OR (area under the curve):ti,ab,kw OR (auc):ti,ab,kw
#5 (glioma*):ti,ab,kw OR (glioblastoma*):ti,ab,kw OR (astrocytoma*):ti,ab,kw
#6 #1 NOT #2
#7 #3 AND #5
#8 #4 AND #7
#9 #6 AND #8

Supplementary Fig. 1 Hierarchical summary receiver operating characteristic (HSROC) curves of different sample sizes, related to Table 4. A.) sample size less than 130 (17 studies). B.) sample size more than 130 (16 studies).

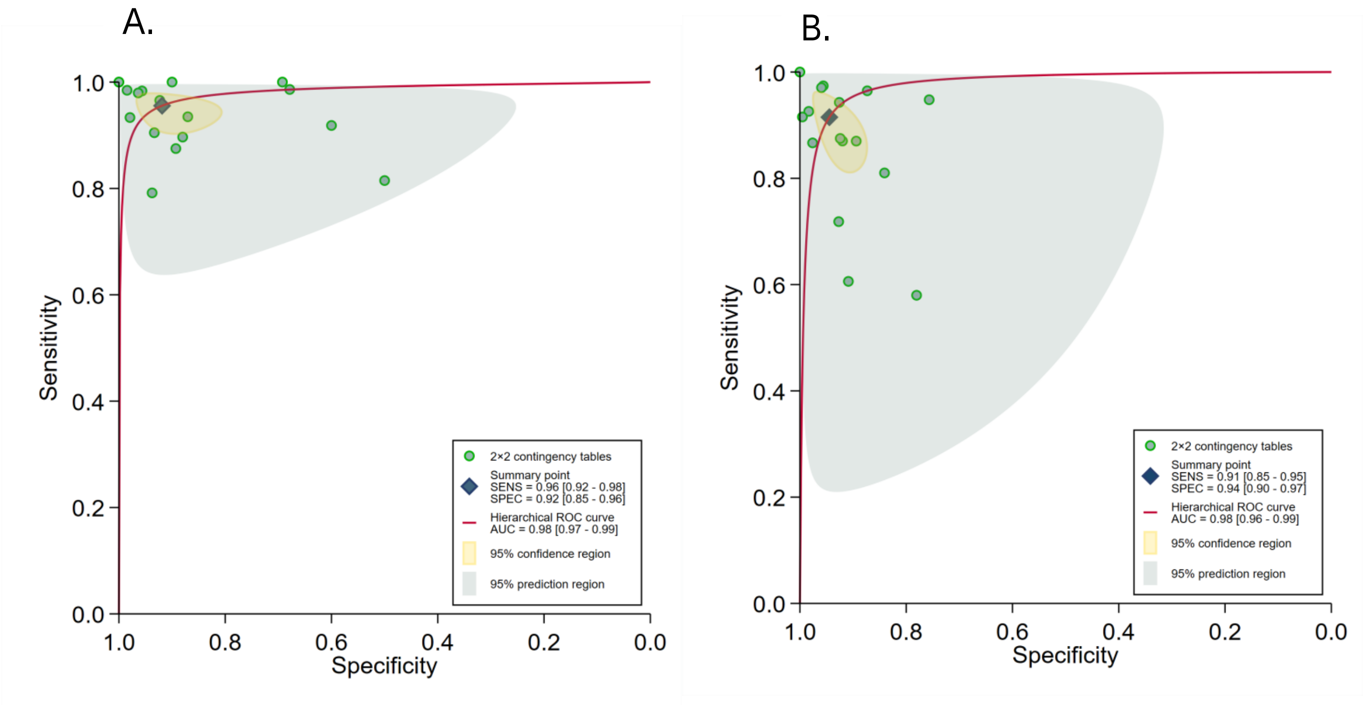

Supplementary Fig. 2 HSROC curves of open access data or not, related to Table 4. A.) open access (26 studies). B.) no open access (7 studies).

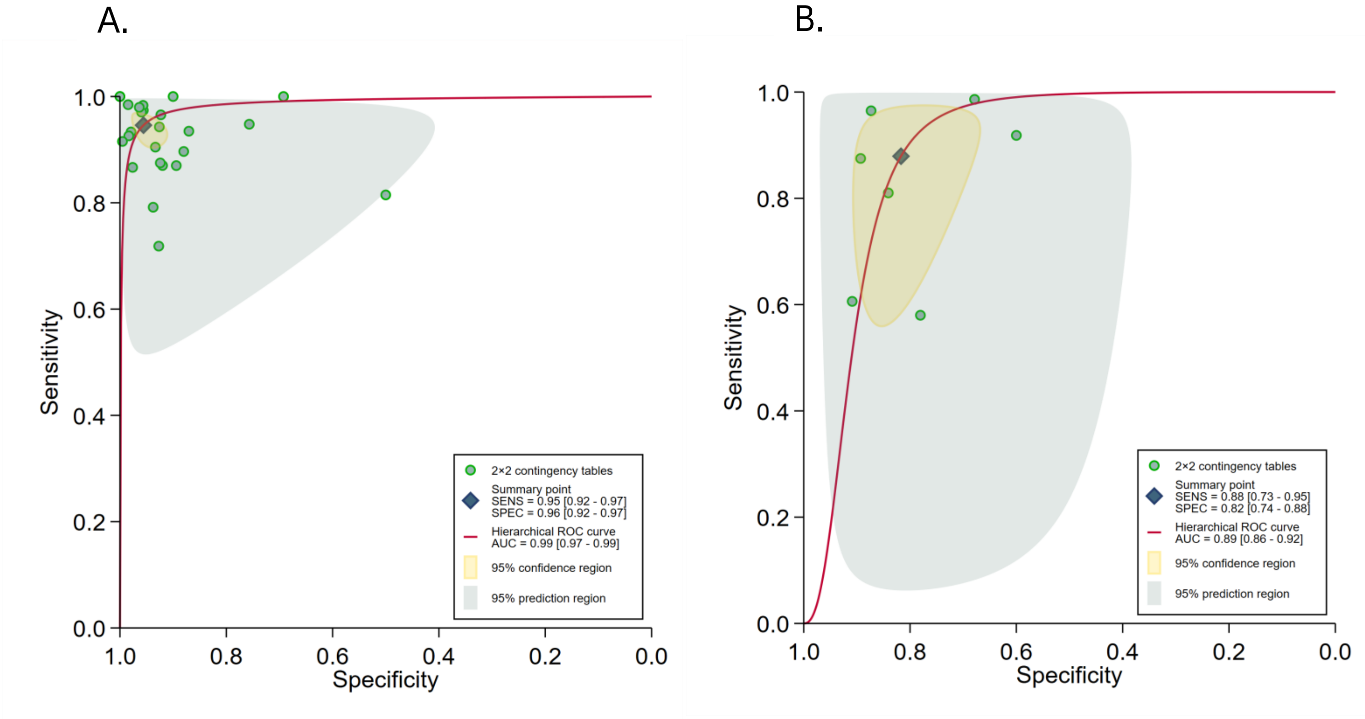

Supplementary Fig. 3 HSROC curves of different internal validation types, related to Table 4. A.) K-fold cross-validation (9 studies). B.) random split-sample validation (24 studies).

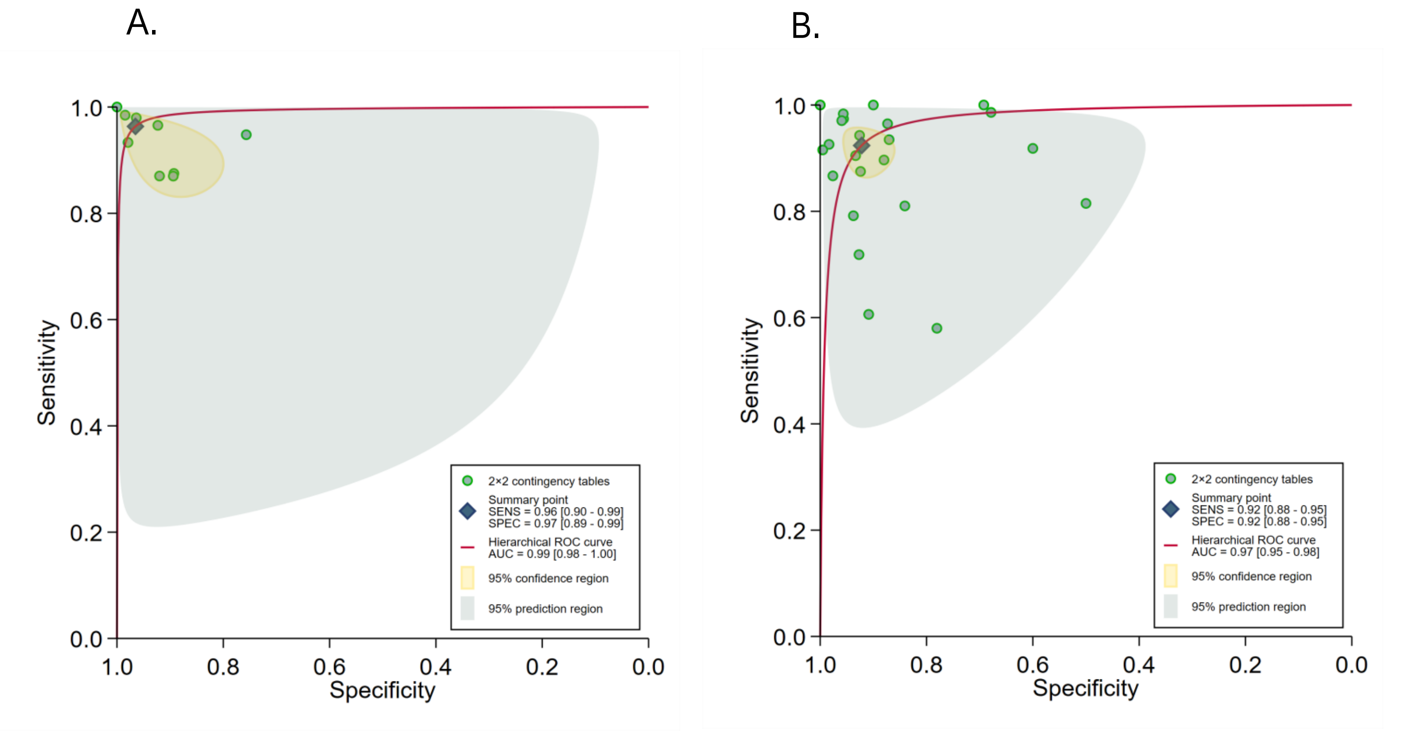

Supplementary Fig. 4 HSROC curves of using transfer learning or not , related to Table 4. A.) transfer learning (9 studies). B .) no transfer learning (24 studies).

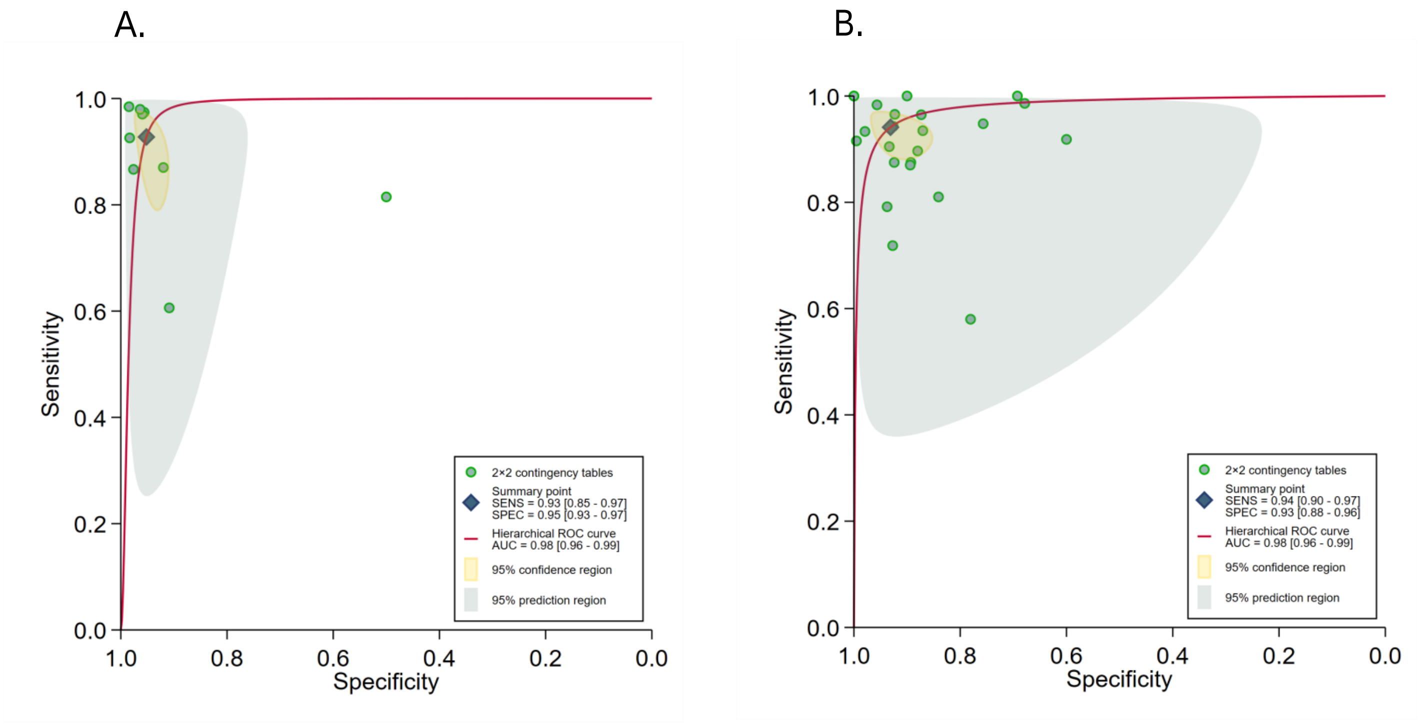

Supplementary Fig. 5 HSROC curves of different data units , related to Table 4.
A.)image-based (20 studies). B.)case-based (13 studies).

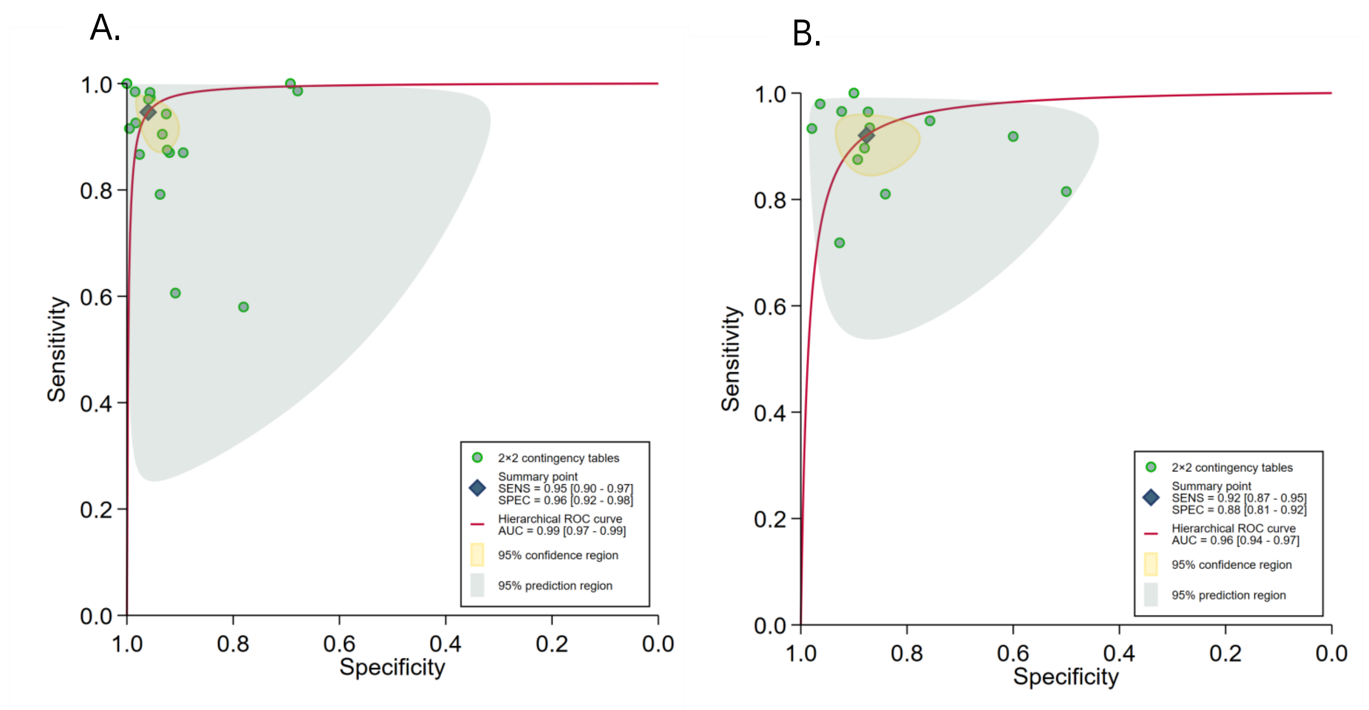

Supplementary Fig. 6 HSROC curves of glioma classification types, related to Table 4.
A .) only grade IV considered high grade glioma (30 tables). B.) grade III and grade IV considered high grade glioma (23 tables).

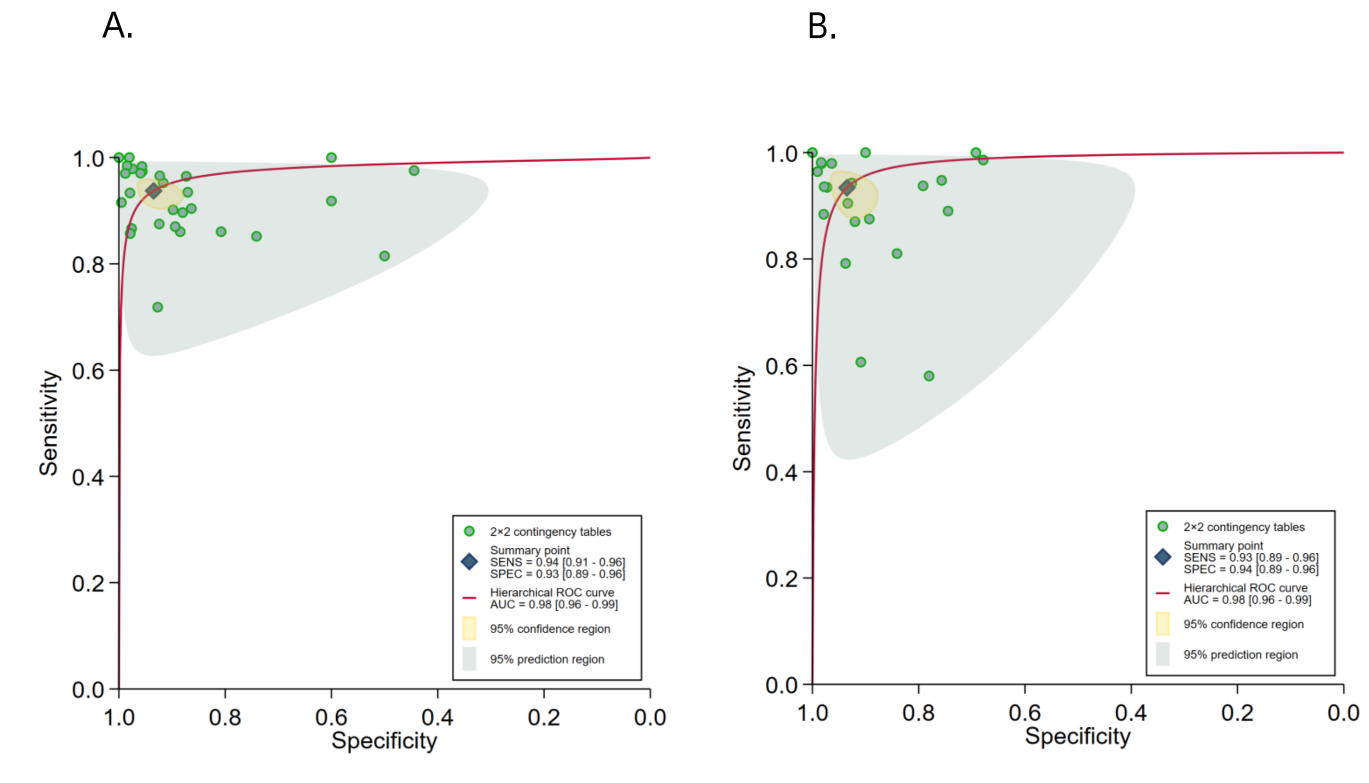

Supplementary Fig. 7 HSROC curves of validation types, related to Table 4.
A.) internal validation (46 tables). B.) external validation (7 tables).

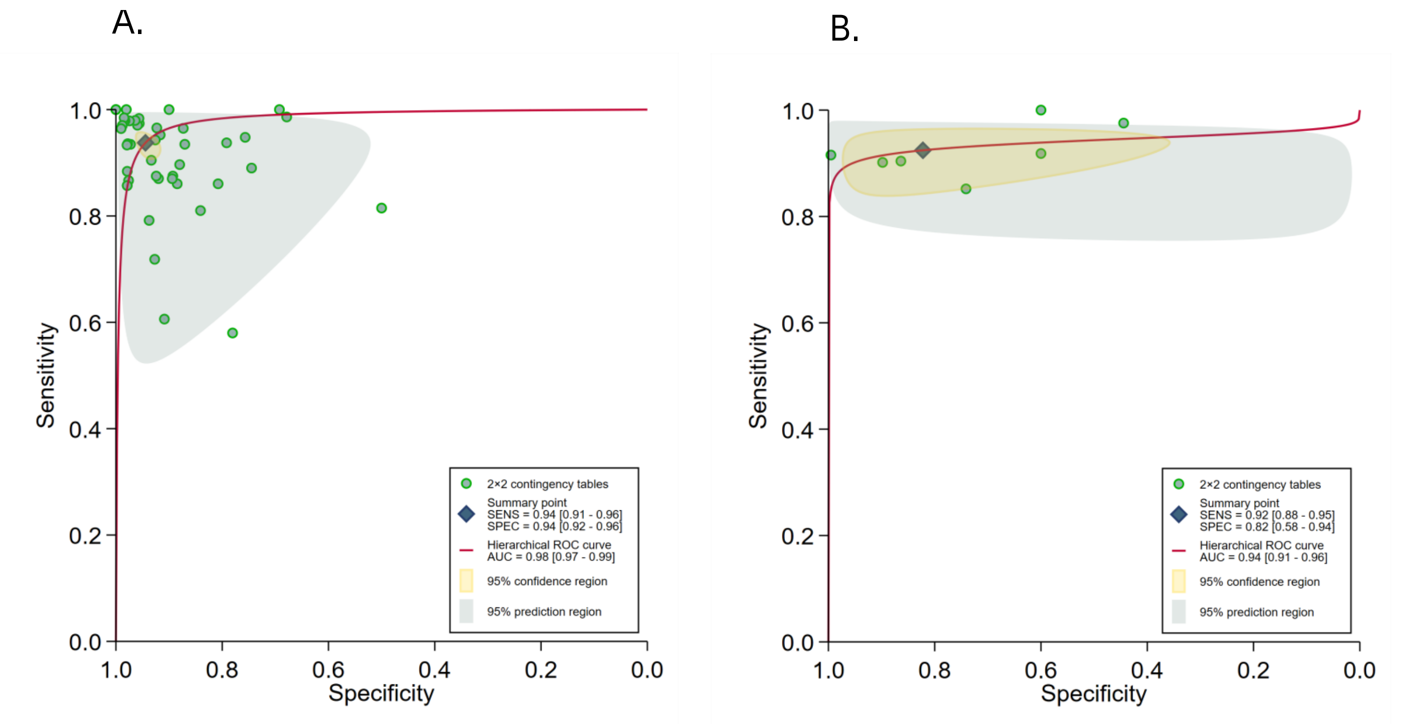

Supplementary Fig. 8 Forest plot of different sample sizes, related to Table 4.
A.) sample size less than 130 (17 studies). B.) sample size more than 130 (16 studies).

A.

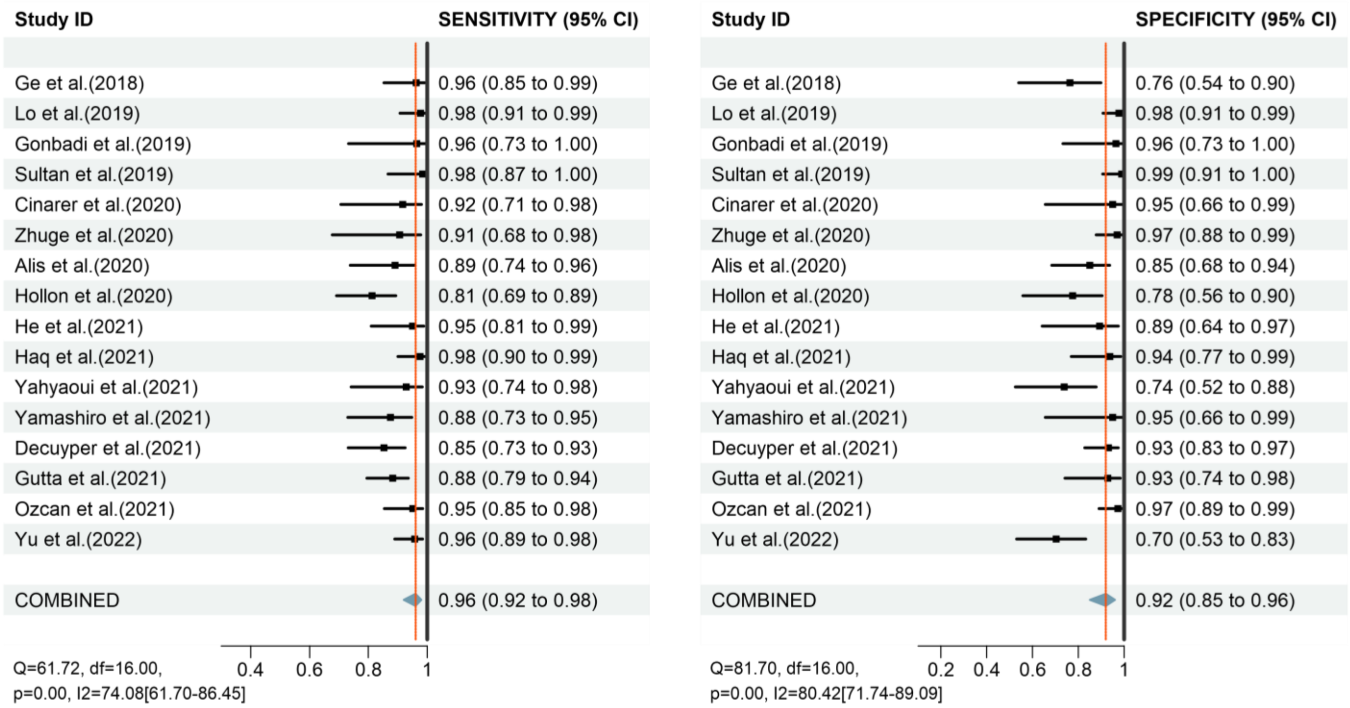

B.

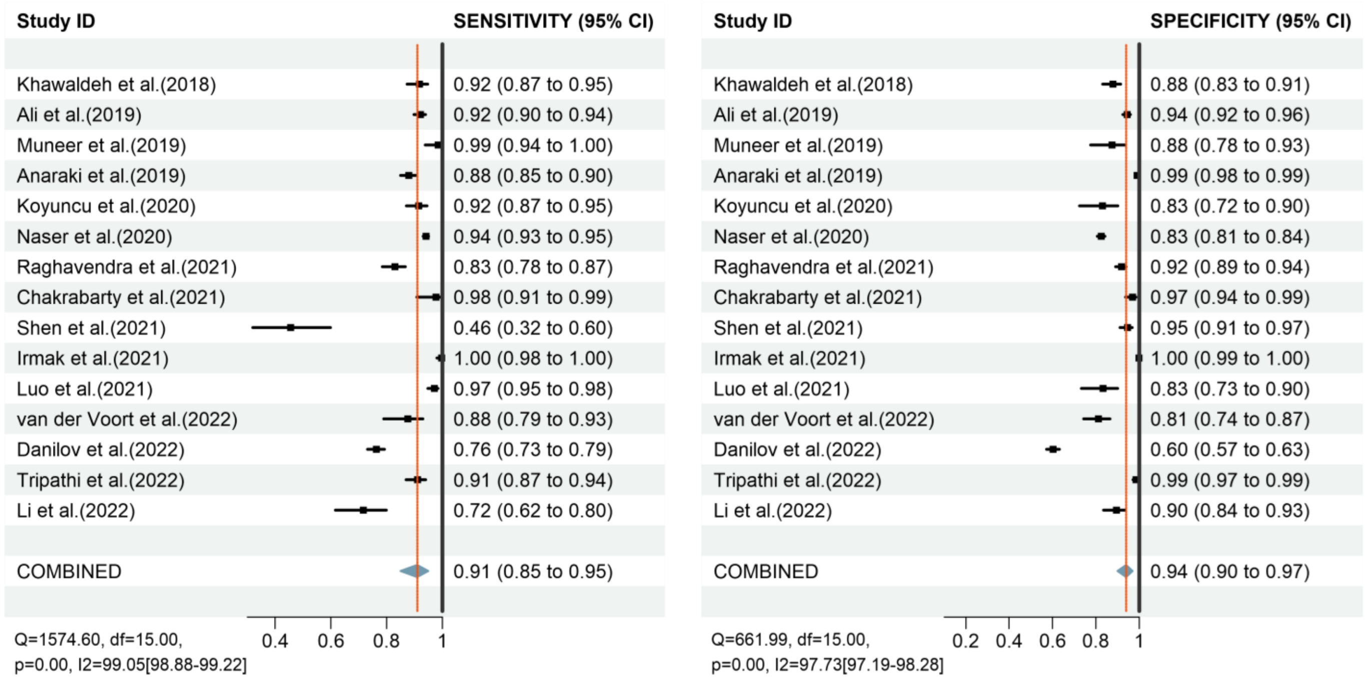

Supplementary Fig. 9 Forest plot of open access data or not, related to Table 4.
A.) open access (26 studies). B.) no open access (7 studies).

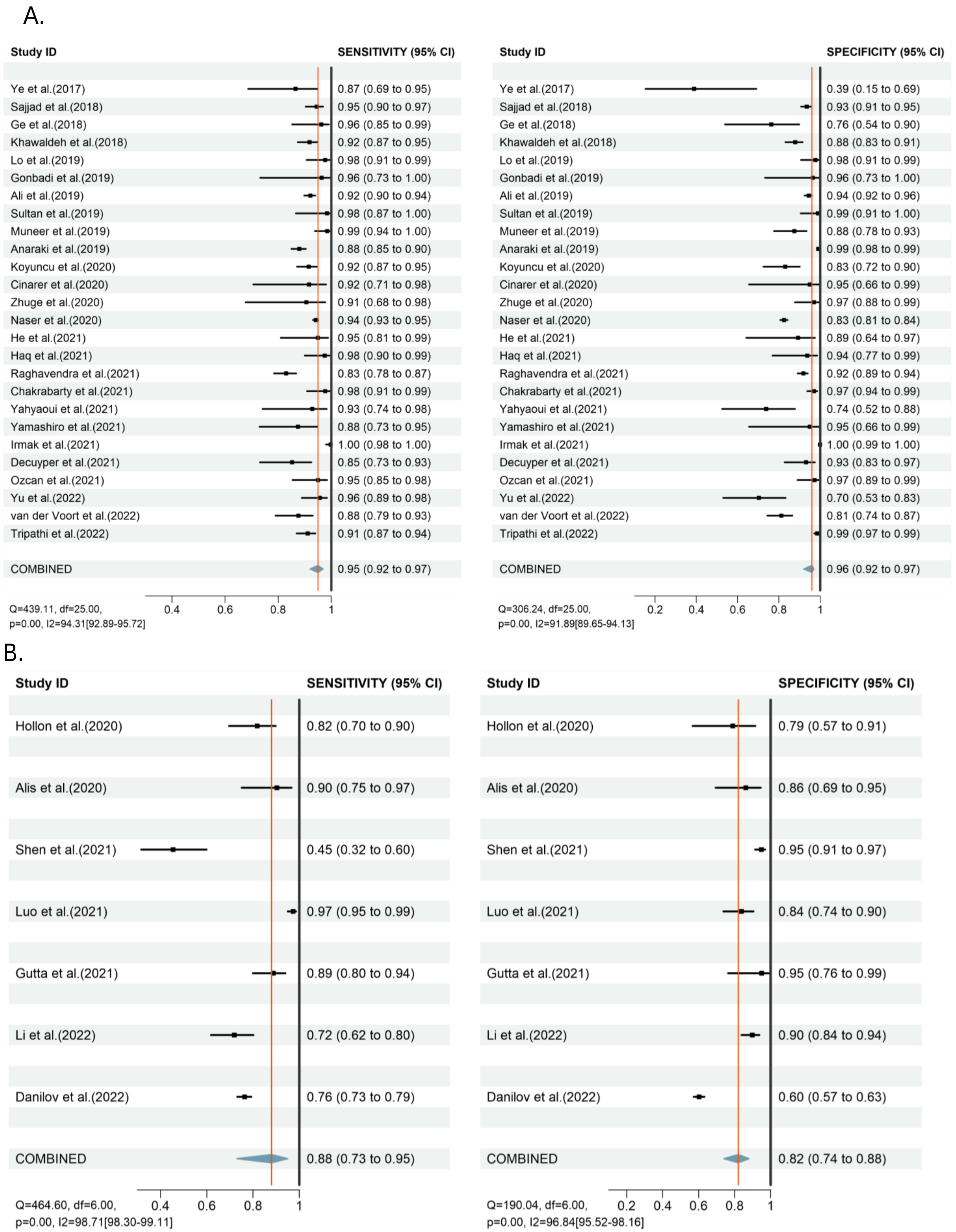

Supplementary Fig. 10 Forest plot of internal validation type, related to Table 4.

A.) K-fold and leave-one-out cross validation (9 studies). B.)random split-sample validation (24 studies)

.

a

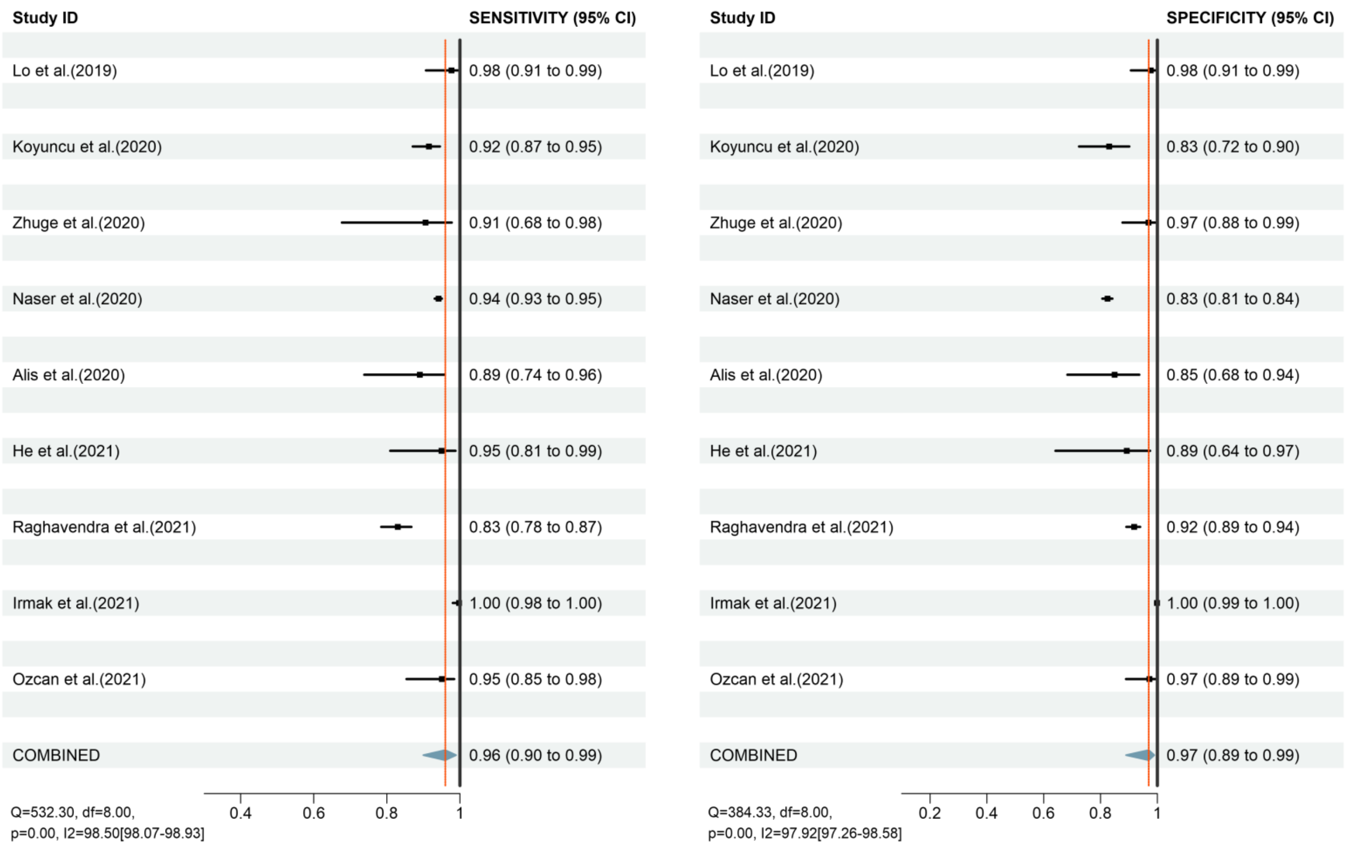

b

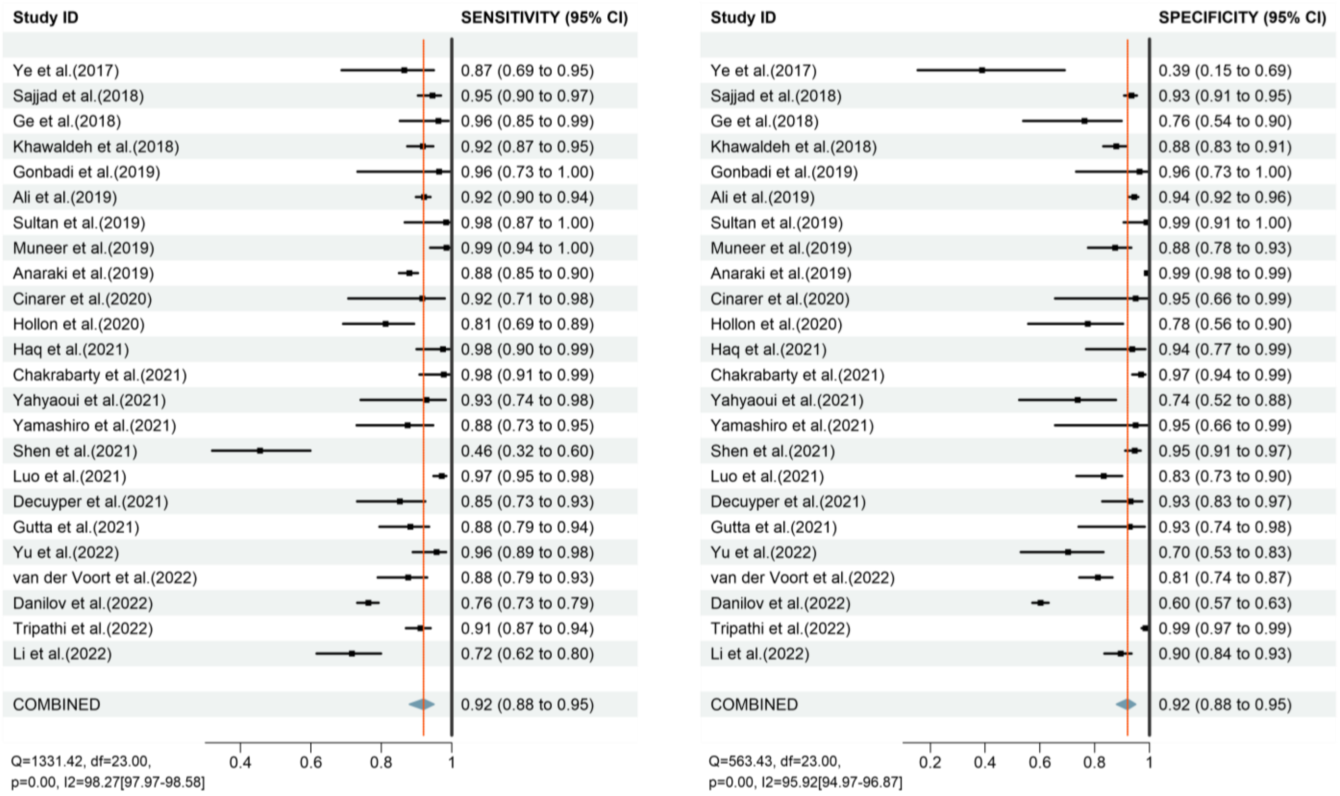

Supplementary Fig. 11 Forest plot of using transfer learning or not, related to Table 4.

A.) transfer learning (9 studies). B.) no transfer learning (24 studies).

A.

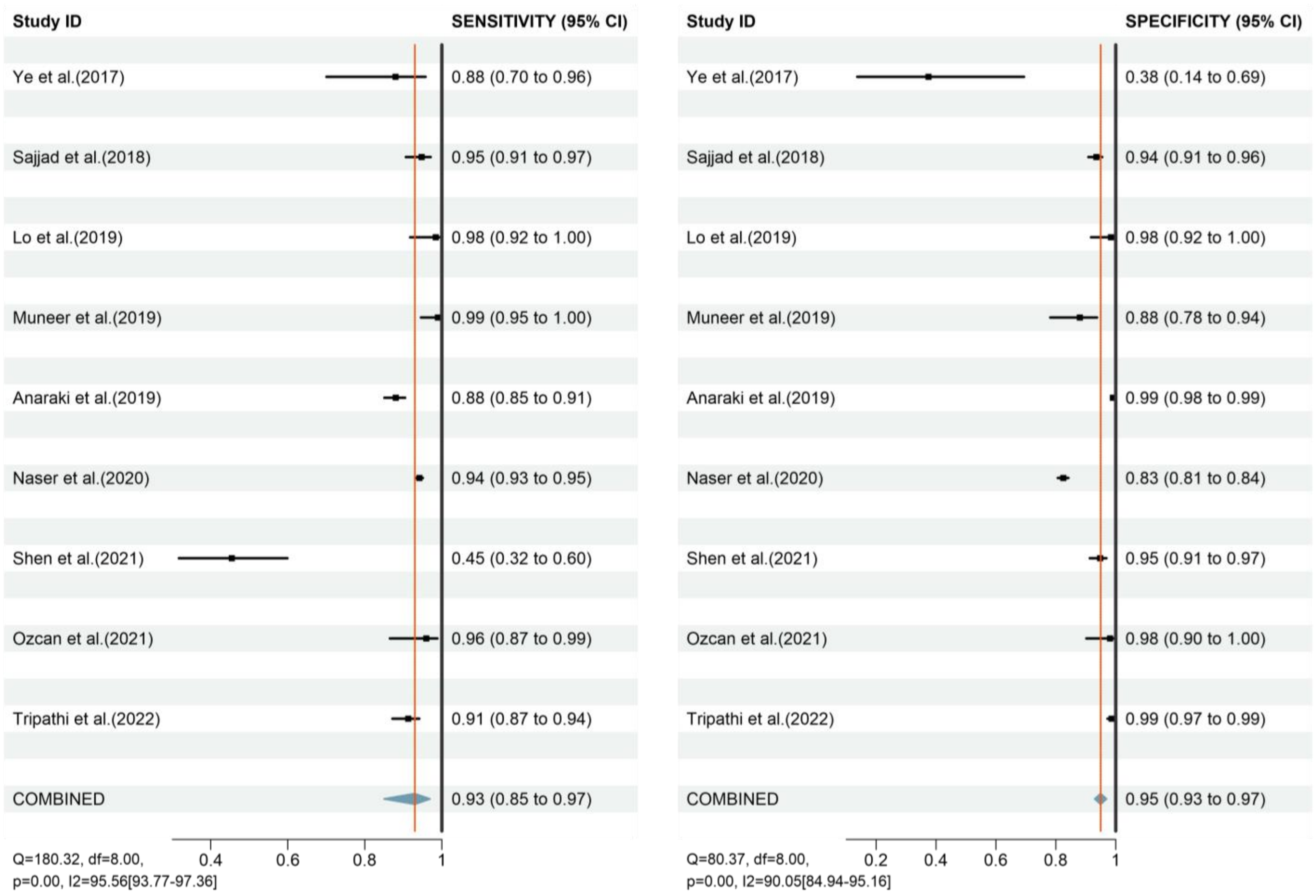

B.

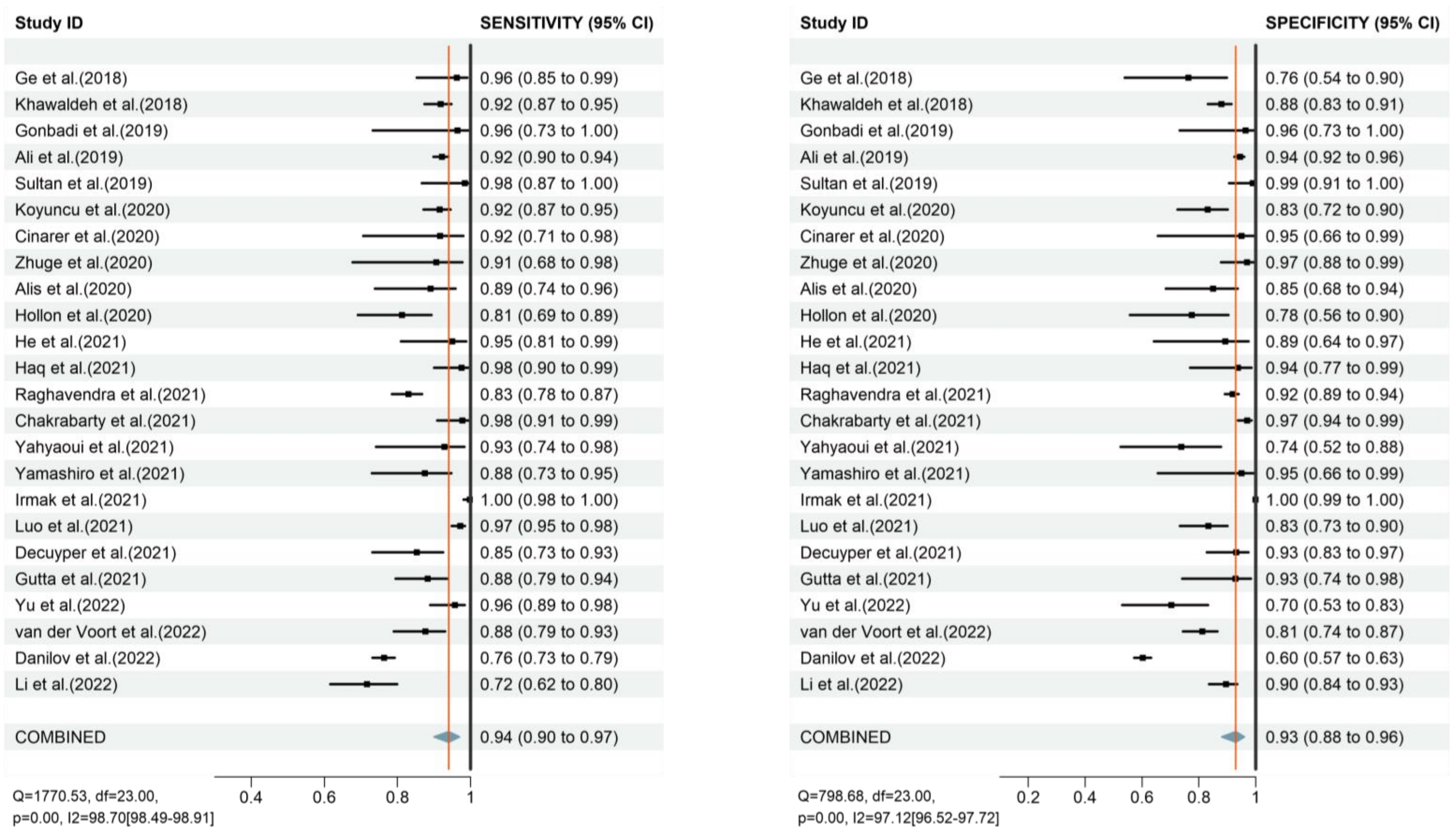

Supplementary Fig. 12 Forest plot of data unit, related to Table 4.
A.) image-based (20 studies). B.) case-based (13 studies).

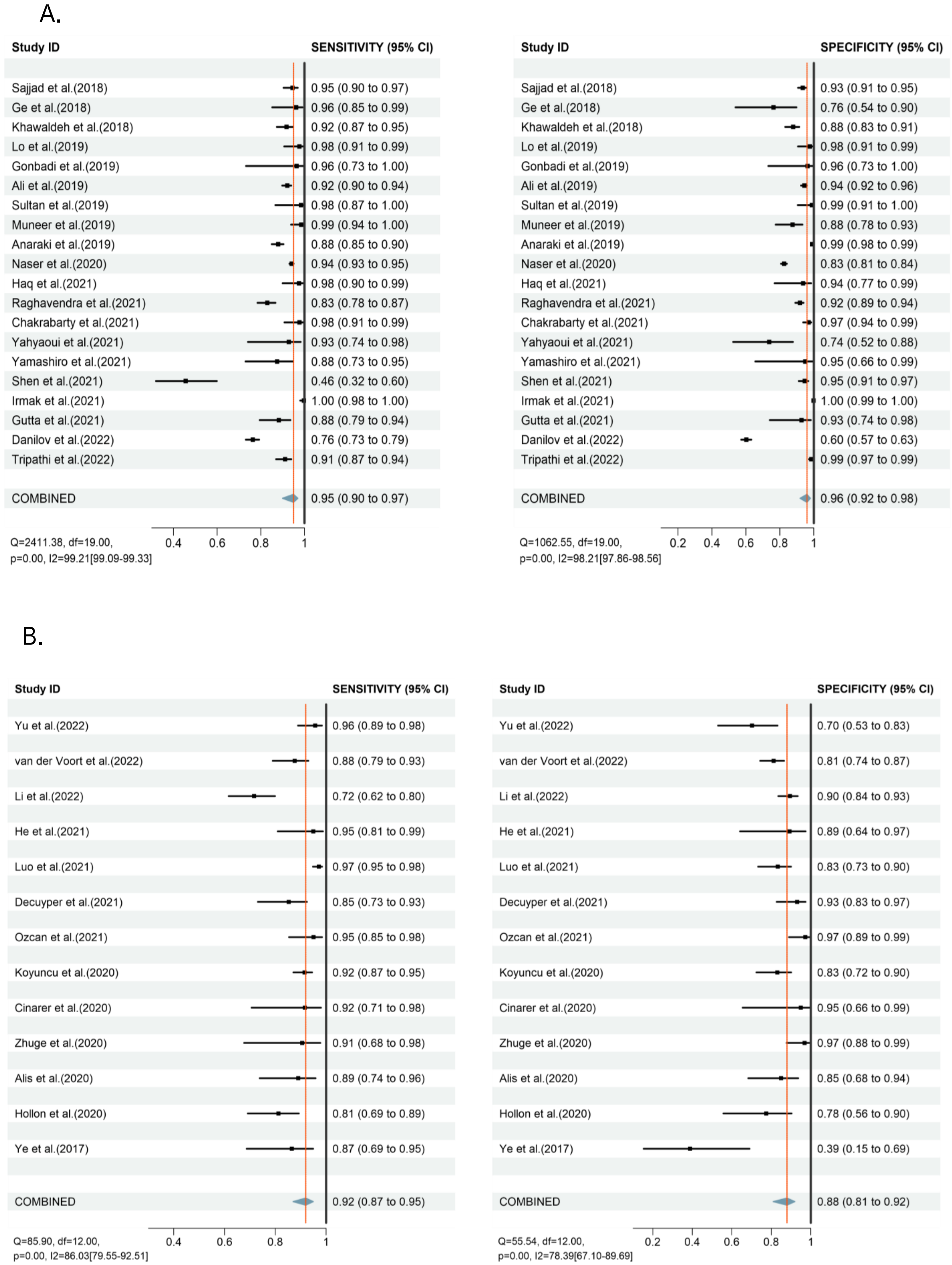

Supplementary Fig. 13 Forest plot of glioma classification types, related to Table 4.
A.)only grade IV considered high grade glioma (30 tables). B.) grade III and grade IV considered high grade glioma (23 tables).

A

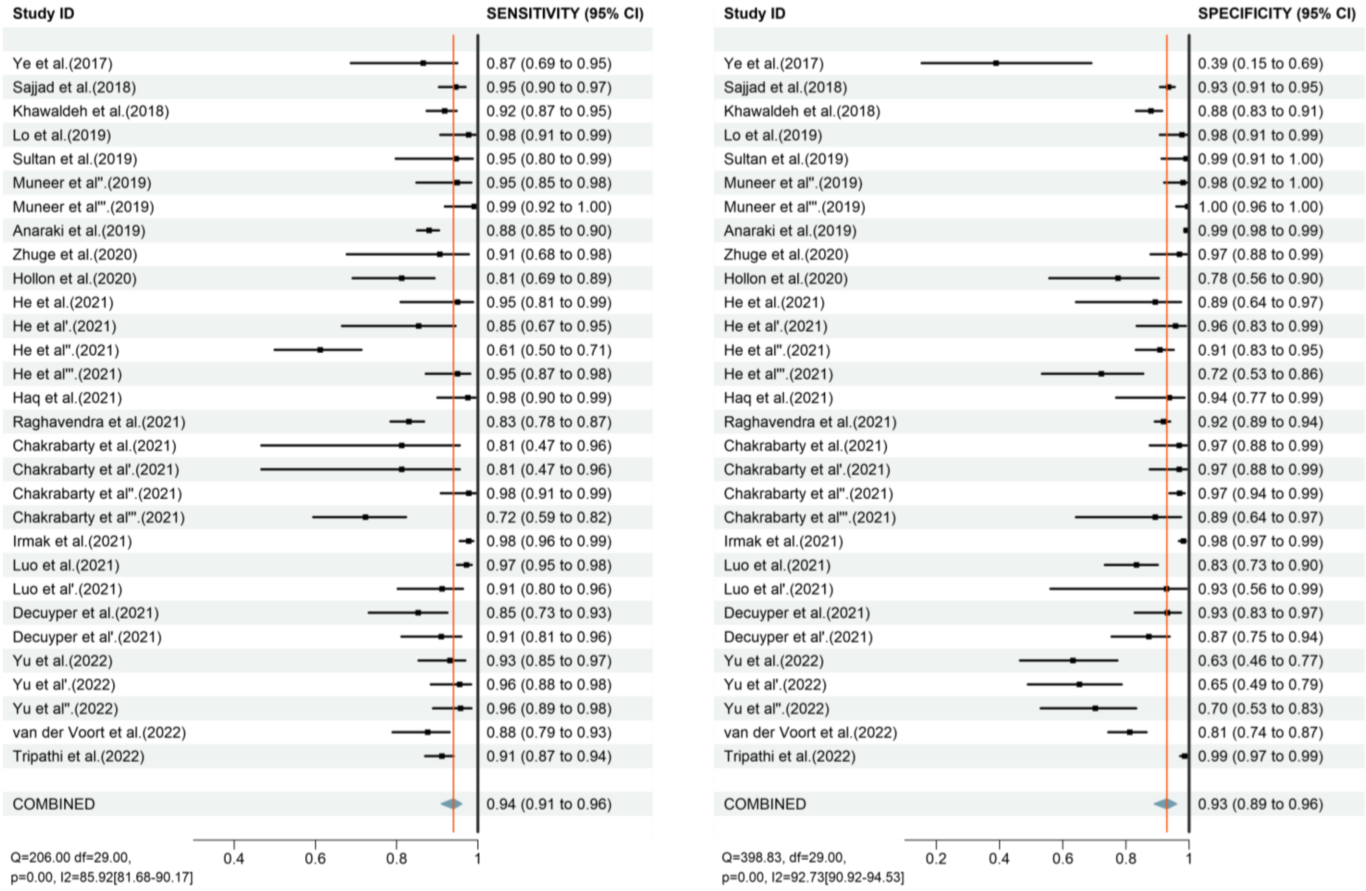

B.

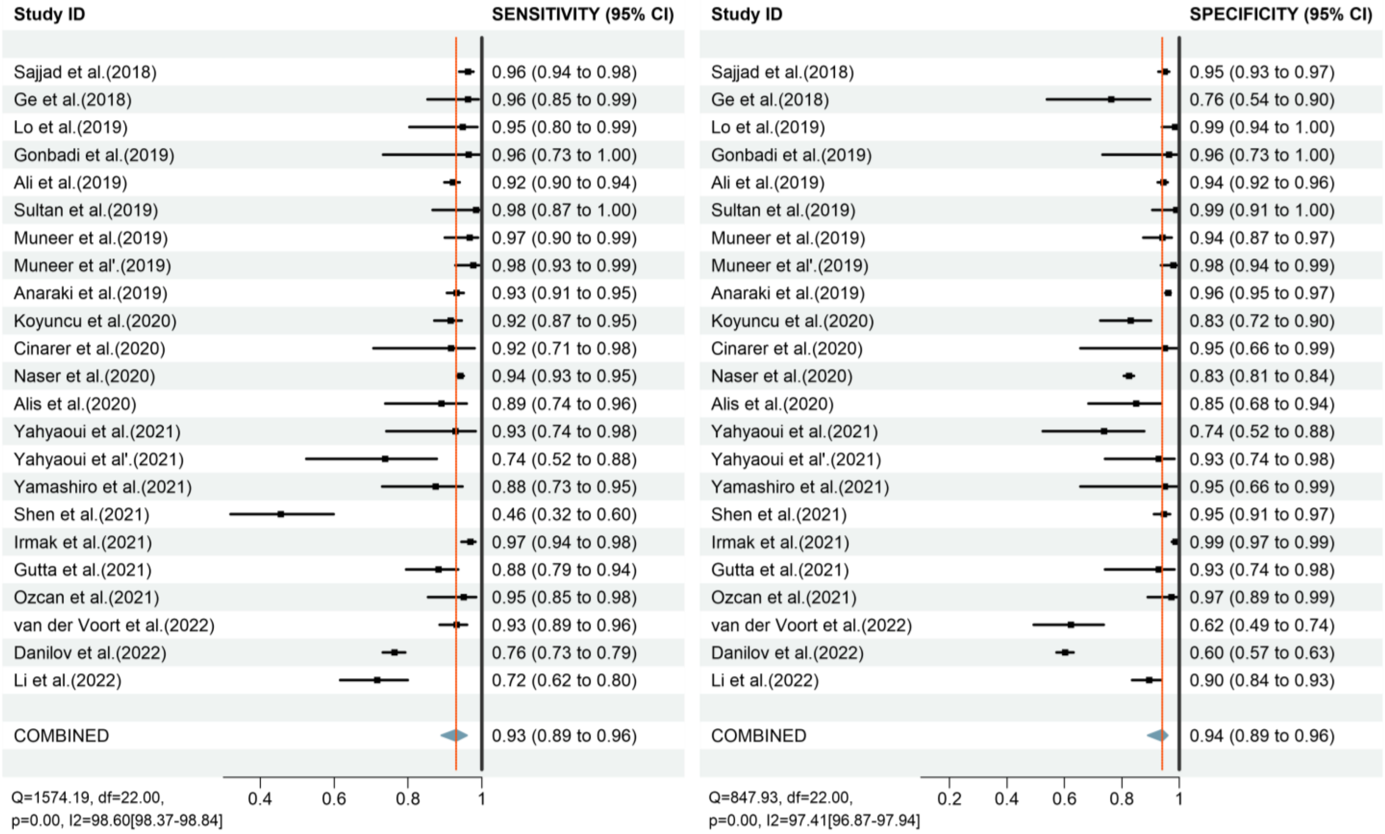

Supplementary Fig. 14 Forest plot of validation types, related to Table 4.
A.) internal validation (46 tables). B.) external validation (7 tables).

A.

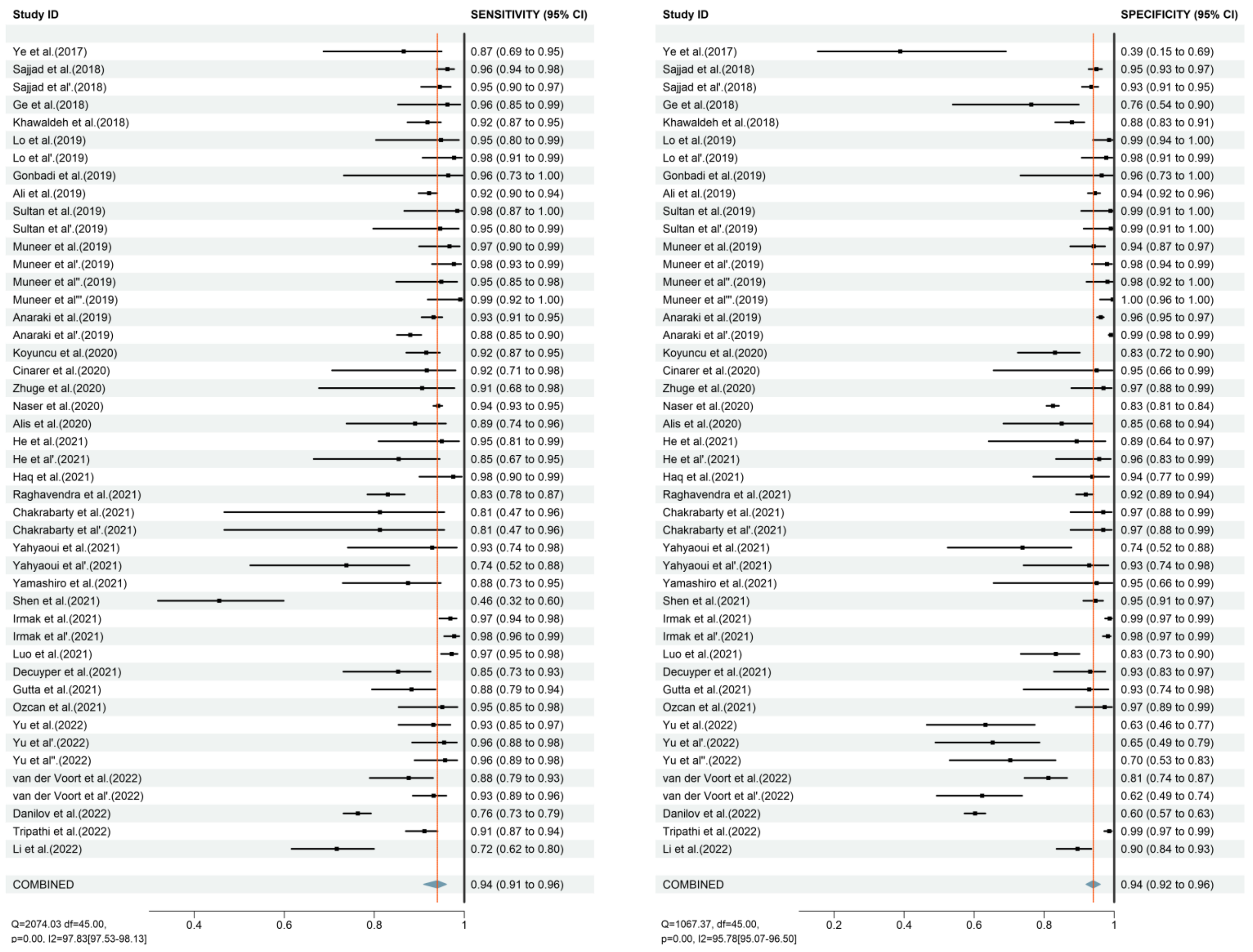

B.

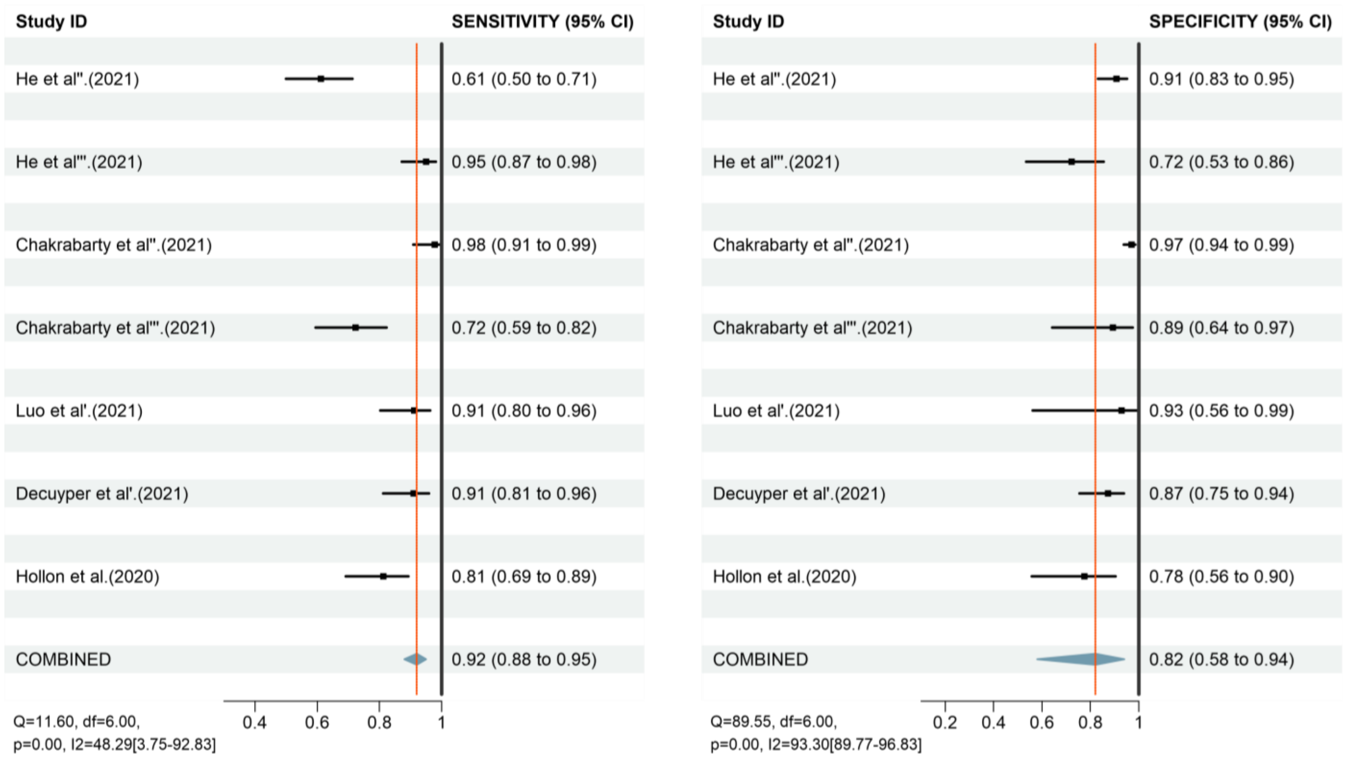

Supplementary Fig. 15 Funnel plot of all studies and studies only reporting the highest accuracy to assess publication bias, related to Table 4. A). All studies. B .) studies reporting the highest accuracy.

A.

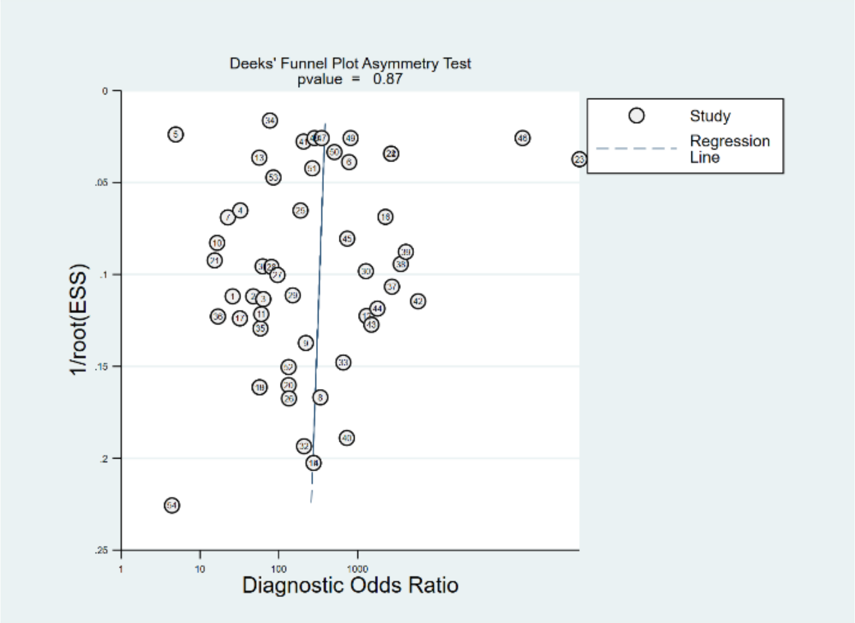

B.

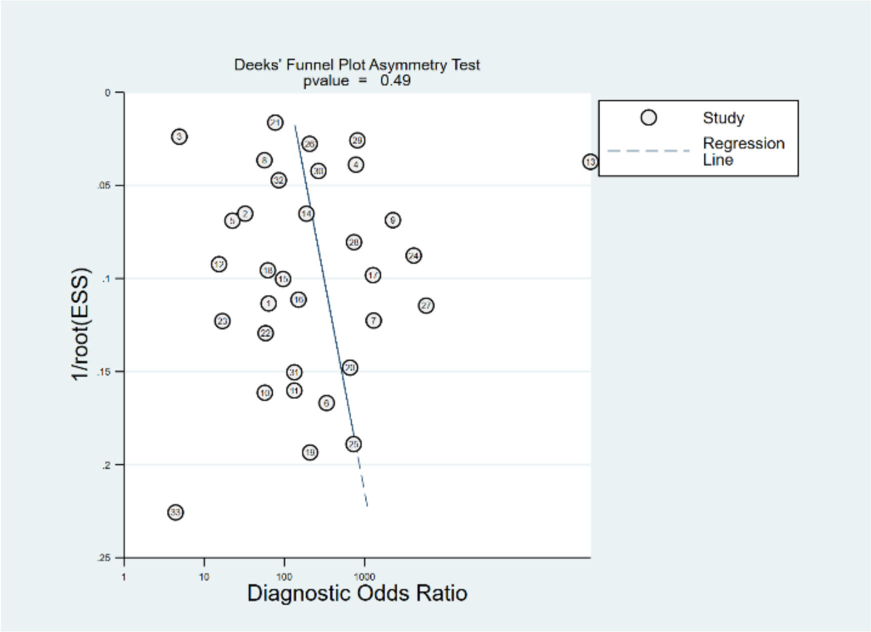

Supplementary Fig. 16 QUADAS-2 summary plot, related to Table 4. Risk of bias and applicability concerns were summarized across all 49 studies.

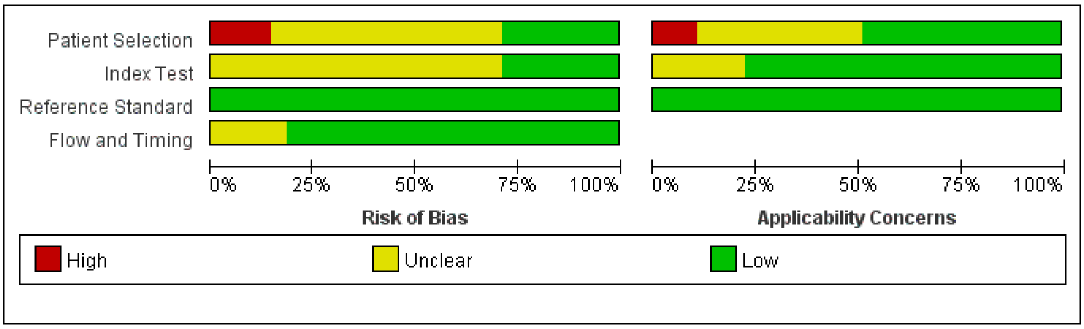

Supplementary Fig. 17 QUADAS-2 plot for each detailed item, related to Table 4.

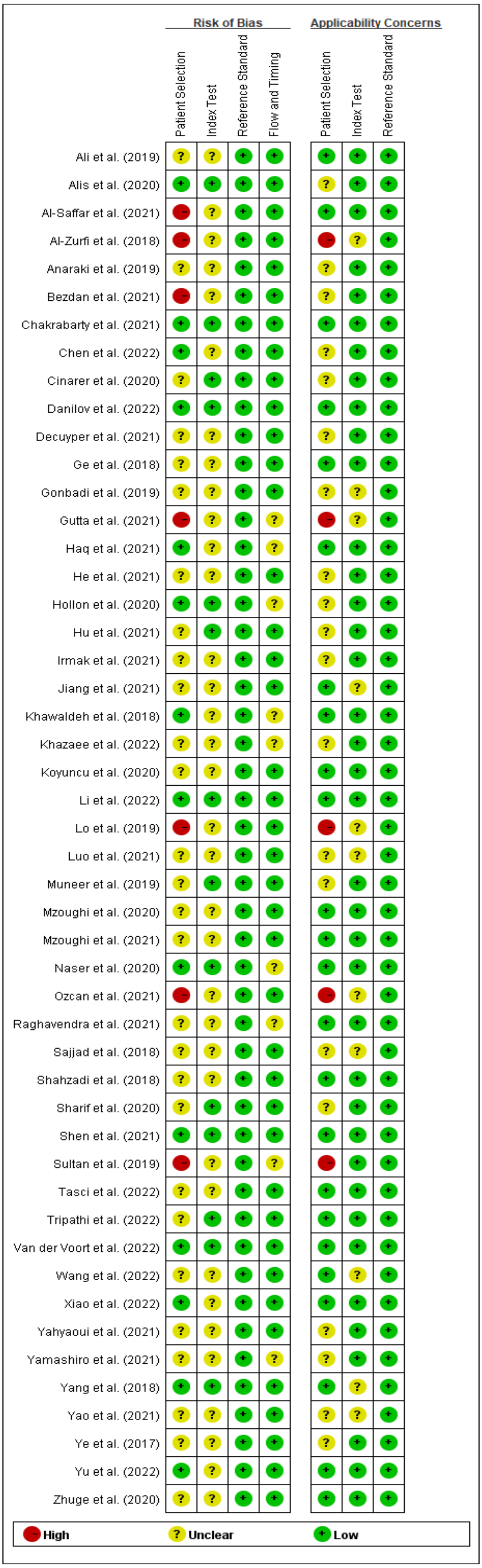

Supplementary Table. 1 Meta regression result, related to Table 4.

Covariates	Subgroup	Number of studies	Sensitivity	P value	Specificity	P value
Sample size	Yes	16	0.91	<0.01	0.94	0.03
	No	17	0.96		0.92	
Data sharing	Yes	26	0.95	0.39	0.95	0.91
	No	7	0.87		0.82	
Internal validation type	No random split	24	0.92	<0.01	0.92	<0.01
	Random split	9	0.96		0.96	
Transfer learning	Yes	9	0.93	<0.01	0.95	0.07
	No	24	0.94		0.93	
Data unit	Image-based	13	0.93	<0.01	0.88	<0.01
	Case-based	20	0.94		0.96	
Classification type	Grade IV High grade	23	0.93	<0.01	0.94	<0.01
	Grade III and IV high grade	30	0.94		0.93	
Validation type	Internal validation	7	0.93	<0.01	0.82	<0.01
	External validation	46	0.94		0.94	
